# A novel PDX modeling strategy and its application in metabolomics study for malignant pleural mesothelioma

**DOI:** 10.1186/s12885-021-08980-5

**Published:** 2021-11-17

**Authors:** Zhongjian Chen, Chenxi Yang, Zhenying Guo, Siyu Song, Yun Gao, Ding Wang, Weimin Mao, Junping Liu

**Affiliations:** 1 The Cancer Research Institute, The Cancer Hospital of the University of Chinese Academy of Sciences (Zhejiang Cancer Hospital), Institute of Cancer and Basic Medicine (ICBM), Chinese Academy of Sciences, Zhejiang, 310022 Hangzhou China; 2 Key Laboratory Diagnosis and Treatment Technology on Thoracic Oncology, Zhejiang, 310022 Hangzhou China

**Keywords:** Patient-derived xenograft, Malignant pleural mesothelioma, GC-MS, Metabolomics, Ultrasound-guided biopsy

## Abstract

**Background:**

Malignant pleural mesothelioma (MPM) is a rare and aggressive carcinoma located in pleural cavity. Due to lack of effective diagnostic biomarkers and therapeutic targets in MPM, the prognosis is extremely poor. Because of difficulties in sample extraction, and the high rate of misdiagnosis, MPM is rarely studied. Therefore, novel modeling methodology is crucially needed to facilitate MPM research.

**Methods:**

A novel patient-derived xenograft (PDX) modeling strategy was designed, which included preliminary screening of patients with pleural thickening using computerized tomography (CT) scan, further reviewing history of disease and imaging by a senior sonographer as well as histopathological analysis by a senior pathologist, and PDX model construction using ultrasound-guided pleural biopsy from MPM patients. Gas chromatography-mass spectrometry-based metabolomics was further utilized for investigating circulating metabolic features of the PDX models. Univariate and multivariate analysis, and pathway analysis were performed to explore the differential metabolites, enriched metabolism pathways and potential metabolic targets.

**Results:**

After screening using our strategy, 5 out of 116 patients were confirmed to be MPM, and their specimens were used for modeling. Two PDX models were established successfully. Metabolomics analysis revealed significant metabolic shifts in PDX models, such as dysregulations in amino acid metabolism, TCA cycle and glycolysis, and nucleotide metabolism.

**Conclusions:**

To sum up, we suggested a novel modeling strategy that may facilitate specimen availability for MM research, and by applying metabolomics in this model, several metabolic features were identified, whereas future studies with large sample size are needed.

**Supplementary Information:**

The online version contains supplementary material available at 10.1186/s12885-021-08980-5.

## Introduction

Malignant mesothelioma (MM) is a rare cancer originating from the mesothelial linings of the pleural or peritoneal cavities. Malignant pleural mesothelioma (MPM) is the predominant form of MM. The incidence of MPM is very low in China (1.5/1000,000) [1]. Unfortunately, the prognosis of MM is extremely poor (survival time of 12-22 months) due to frequent late diagnosis that results from difficulties in early detection and lack in efficacy of current treatments [2]. Thus, MPM has been a dismal disease troubling both patients and clinical doctors.

MPM is normally induced by asbestos [3]. Although this mineral fiber has been widely banned globally, it is still consumed in several countries, such as India, Russia, and China [4]. Thus, MPM will continue to burden society due to previous and continuous use of asbestos as well as the long latency (20-40 years) of MPM [5]. However, most doctors and pathologists in China are unfamiliar with MM due to its rarity, leading to a delay in treatment and even misdiagnosis, denoting an urgent need in identification of diagnostic biomarkers [6].

Lack of specimens is the forefront obstacle in MPM research in China, even to the whole globe. Two studies suggested that the patient-derived xenograft (PDX) model, which implants tumor tissue from a patient into recipient mice, is suitable for testing both anti-cancer therapies and the biological functions of genes or proteins in MM [7, 8]. Compared to cell line-derived xenograft, PDX model better simulates clinical samples, because it greatly reserves heterogeneity of the primary tumor [8]. Accordingly, PDX modeling is efficient and advantageous for biomarker detection and drug screening.

Construction of PDX models relies on specimens from patients, which are commonly collected from surgery. However, in China, surgery in MPM is infrequent thus can rarely provide samples for modeling. Ultrasound-guided (US-guided) biopsy is often used for examination of unclear malignancies, and it is still able to provide samples under situations when MPM patients are not suitable for surgery (e.g., pleural adhesions) [9]. Therefore, this study established a novel PDX modeling strategy based on an US-guided pleural biopsy, attributable to cooperation across three specializations, including US imaging, pathology, and lab research. Success in the establishment of two PDX models indicates the feasibility of our strategy.

To date, many studies focus on genomes in MM [10–12], with relatively fewer investigating metabolomes. Nevertheless, metabolome has been viewed to utmost demonstrate phenotypes, indicating its importance and prominence for disease exploration through the discovery of metabolic biomarkers and therapeutic targets [13]. Bononi et al. reported that MM initiation is linked to metabolic reprogramming [14], and Zhang et al. also revealed that metabolic enzymes, such as SLC7A11, might serve as treatment targets for MM [15]. For the above reasons, we aimed to detect diagnostic metabolites and discover biological targets for MPM, through serum-based metabolomic profiling for the US-guided pleural biopsy-derived PDX model.

## Materials and methods

### Ethical statement

This study was performed in accordance with the Declaration of Helsinki (revised in 2013), and the protocol was approved by The Ethical Committee of Zhejiang Cancer Hospital (approval number: IRB-2018-9). Informed consent was obtained from each patient.

### Patients screening for PDX construction

Patients who displayed pleural thickening in computed tomography (CT) imaging were reviewed by an experienced sonographer (Junping Liu) between March 1^st^ 2018 and December 30^th^ 2019 in Zhejiang Cancer Hospital. After exclusion, US-guided pleural biopsy was performed for the remaining patients. Those from patients who did not meet the following exclusion criteria were highly suspected (by the sonographer based on his past clinical experiences) to be MM: 1) pleural metastasis from lung cancer; 2) pleural metastasis from other cancers; 3) tuberculosis lesions; 4) unclear diagnosis; and 5) other reasons (according to imaging features and clinical history). Under the premise of not interfering with clinical diagnosis, these biopsies were collected and implanted into immunodeficient mice for constructing PDX models. At the same time, all biopsies were sent to a senior pathologist (Zhenying Guo) for precise diagnosis.

The animal experiment was permitted by the Institutional Animal Care and Ethics Committee of Zhejiang Cancer Hospital (No. 2018–03-054), in compliance with national or institutional guidelines for the care and use of animals. All the animals were euthanized using isoflurane at the end of experiment.

### US-guided pleural biopsy

Patients were fasted for at least 8 h before biopsy. The biopsy was performed according to Zhang et al. [16] Briefly, Esaote MylabTMTwice ultrasound apparatus (Italy) with probes configuration convex transducer CA541 and linear LA524 was utilized. First, a low-frequency probe (4.0 MHz) was used to detect the pleural effusion, pleura and blood flow, and the thickest point of the pleura was selected for biopsy. Second, pleural biopsy was guided through alternatively using low-frequency (4.0 MHz) and high-frequency (9.0 MHz) probes conducted by two experienced sonographers. One sonographer provided the guidance, and the other used an 18 or 16 G automated cutting needle (MC1816, Bard Max. Core, Bard Inc., USA) to perform the biopsy under local anesthesia with 2% lidocaine. Specimens were immediately fixed in 10% formalin and sent to the Department of Pathology for examination. Without interfering clinical diagnosis, part of fresh specimens was sent for constructing PDX models.

### Immunohistochemistry (IHC) staining

IHC staining was performed according to Wu et al. [8] In brief, slides of formalin-fixed and paraffin-embedded tumors were deparaffinized and incubated in 3% hydrogen peroxidase. After inactivation of endogenous peroxidases, slices were treated with antigen retrieval by boiling at 100 °C for 90 s in citric acid repair solution (pH = 6). After blocking, slices were incubated with antibodies overnight at 4 °C. Slices were then incubated with HRP-labeled secondary antibody for 30 min. According to gender, histological features, and location of the tumor, a panel of biomarkers for IHC analysis was selected and the diagnosis was made by a senior pathologist (Zhenying Guo). Details of the antibodies used were listed in the supplemental information.

### PDX model construction

The procedure of PDX construction was similar to established protocol from Wu et al. [8] In brief, 5-week-old female BALB/c immunodeficient mice (certificate number: 2017005004641) were purchased from Shanghai Slac Laboratory Animal Company (Shanghai, China). The mice adapted to the environment for 1 week. Every five of all mice were kept in one cage with free access to food and water, to a 12 h/12 h light/dark cycle, at temperature between 22 °C and 26 °C, with 55% relative humidity. Fresh tumor tissue was kept in a sterilized PBS buffer on ice, and was cut into blocks of 2 × 2 × 2 mm and then was engrafted subcutaneously into the flanks of BALB/c mice (P0) with a trocar needle. A PDX model which can be consecutively passed over twice is defined as a success (P2). Then P2 tumors were harvested and transplanted to 10 mice for the PDX model, and 7 mice without treatment were taken as a control group. When the average tumor size exceeded 200 mm^3^, blood was collected retro-orbitally under isoflurane anesthesia. Sera were separated with centrifugation for 10 min at 2400 g, 4 °C and were kept at -80 °C until analysis.

### Gas chromatography-mass spectrometry (GC-MS)-based metabolomics

The GC-MS-based metabolomics was performed according to the previously published method from Zhao et al. [17] The procedures including sample preparation and GC-MS analysis are described in the supplemental information.

### Metabolomic data analysis

After data formatting (to .abf) with Reifycs Abf Converter (https://www.reifycs.com/AbfConverter/), MS-DIAL software was used for data processing, including peak picking, peak alignment, missing value interpolation, and so on. Metabolite annotation was performed through the untargeted database of GC-MS from Lumingbio. Finally, a data set with sample information and peak information was obtained. Principal component analysis (PCA) and partial least-squares-discriminant analysis (PLS-DA) were performed to visualize the metabolic shift among groups using R package *ropls* (version 1.18.8). Variable importance in the projection (VIP) was obtained from PLS-DA, which ranks the contribution of metabolite features in the PLS-DA model. Finally, features with VIP > 1.0 and *P*-value < 0.05 from two-tailed Student’s t-test were defined as differential metabolites. Heatmaps were plotted to illustrate the metabolic patterns using R package *pheatmap* (version1.0.12). Metabolic pathway analysis was performed using the online tool *Metaboanalyst 5.0* (https://www.metaboanalyst.ca/MetaboAnalyst), and results were visualized using R package *ggplot2* (version 3.3.3). Illustrations of metabolites and their corresponding pathways were created with BioRender (https://biorender.com).

## Results

### Patient information and model construction

**Fig. 1:**
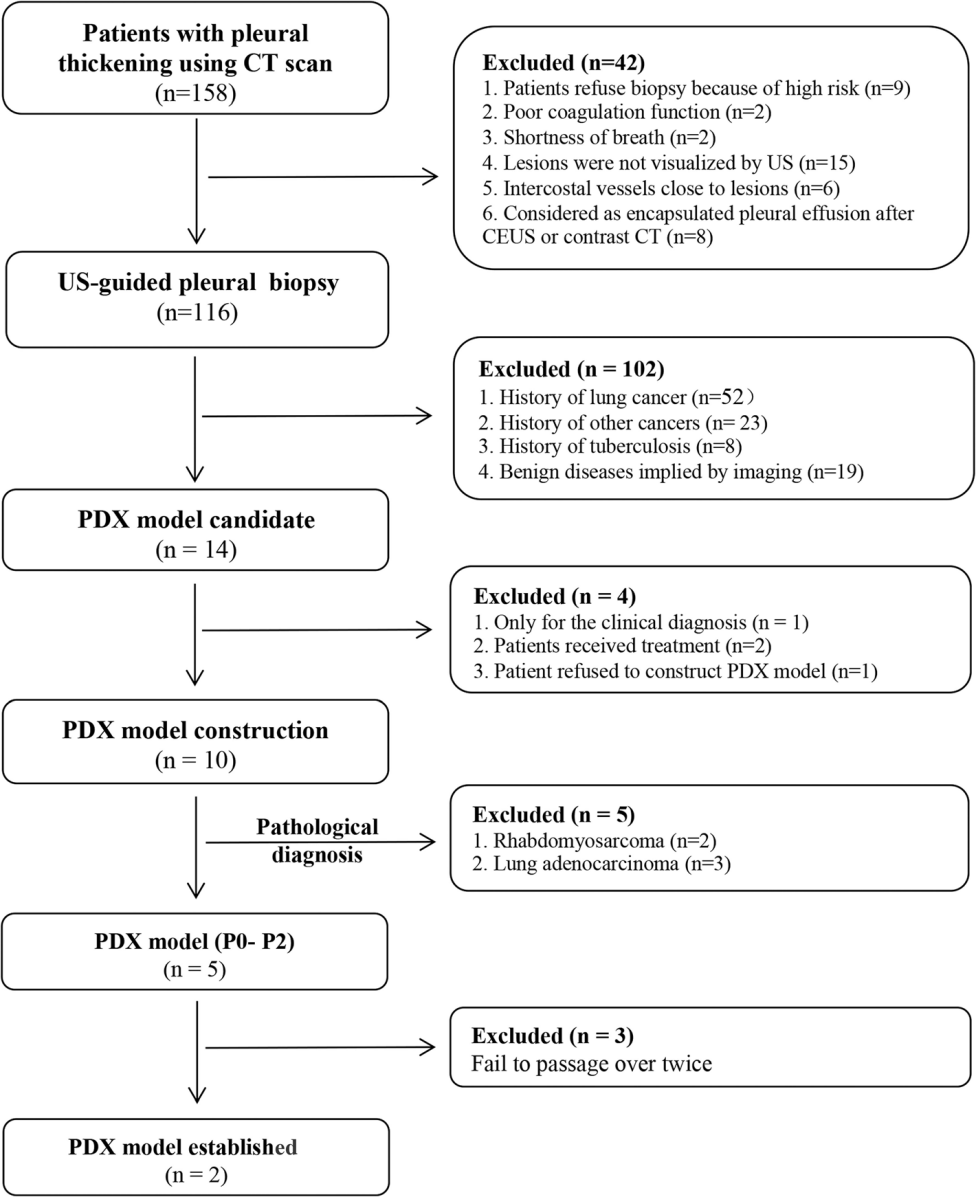
Ultrasound-guided pleural biopsy-based PDX modelling strategy for malignant pleural mesothelioma

**Fig. 2:**
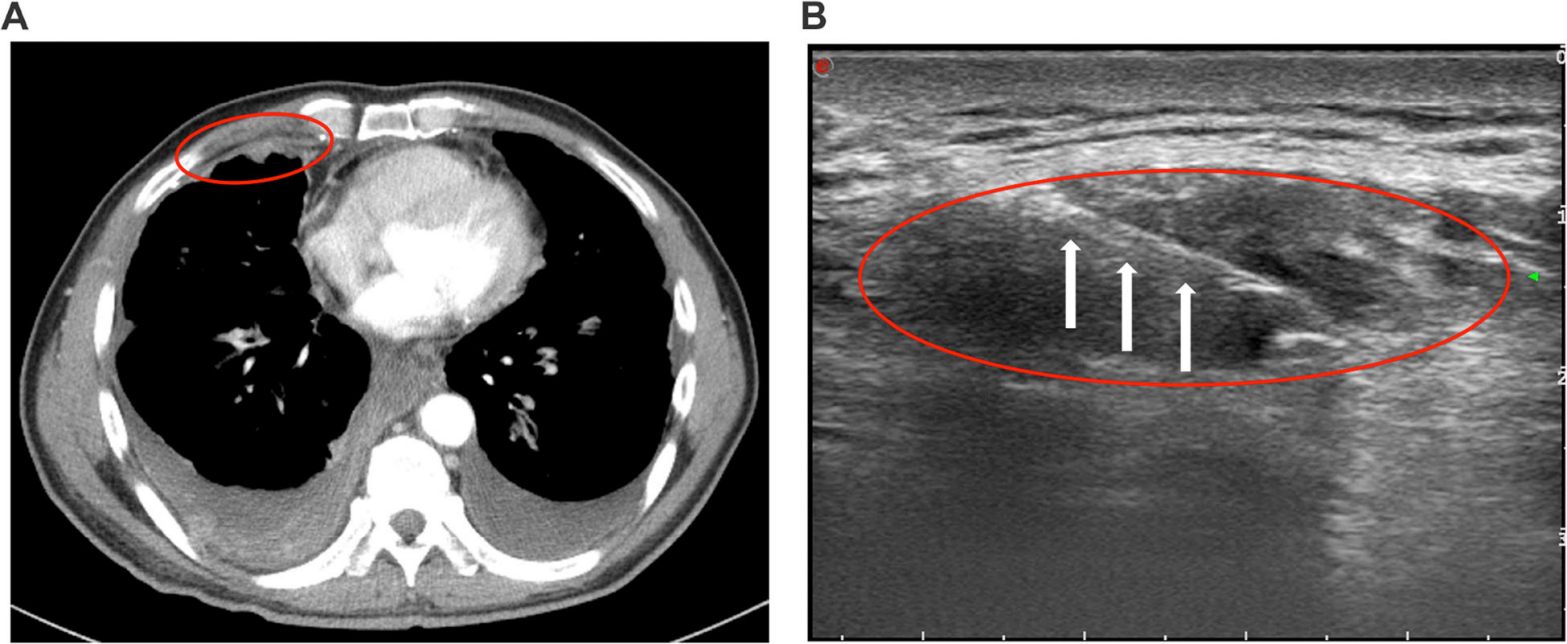
Typical CT scan and ultrasound imaging of patients with pleural thickening. (**A**) CT scan, red oval refers to the pleural thickening area; (**B**) Ultrasound imaging, red oval refers to the pleural mass, white arrows point to the biopsy needle

From March 1^st^ 2018 to December 30^th^ 2019, 158 patients with pleural thickening were reviewed by a senior sonographer (Junping Liu) (Fig. 1). A typical pleural thickening in CT scan was shown in Fig. 2A. A total of 42 individuals were excluded, and the remaining (*n* = 116) patients were performed with US-guided pleural biopsy and pathological diagnosis (Fig. 1, Fig. 2B, Table S1). Fourteen of them were suspected to be MPM (Table S2), among which only 10 biopsies were accessible and were subsequently implanted into mice. Biopsies pathologically diagnosed with other diseases (*n* = 5), including lung adenocarcinoma (*n* = 3) and rhabdomyosarcoma (*n* = 2), were further excluded (Fig. 1). Among the 5 pathologically confirmed MM, 3 failed to grow on mice from P0 to P2, while 2 were successfully constructed. PDX1 was from a 55-year-old male with pleural thickening of annular nodules, lumps in the lungs and chest wall; while PDX2 was from an 85-year-old female, who showed pleural thickening of annular nodules, chest wall lumps. Their detailed information was shown in Table 1.

**Table 1:** Patient characteristics for PDX1 and PDX2 models

Parameters	PDX1	PDX2
Sex	Male	Female
Age (year)	55	85
Body mass index	20	15
Asbestos exposure	No	No
Smoking	No	No
Drinking	Yes	No
Tumor location	Pleural	Pleural
Cell types	Epithelioid	Sarcomatoid
CT scan	Pleural thickening of annular nodules, lumps in the lungs and chest wall	Pleural thickening of annular nodules, chest wall lumps
Family history of tumor	No	Yes
Immunohistochemistry	CK(+), Ki-67(+,70%), TTF1(−), CK5/6(+), WT-1(+), CR\Calretinin(+)	TTF1(−), CK(+), EMA(+), Vim(+), CR\Calretinin(+), D2–40(−), CK5/6(−), WT-1(−), NapsinA(−), P63(−), MyoD1(−), Myogenin(−), S-100(+), CAM5.2(+),SOX10(−), HMB45(−), Melan-A(−), CD31(+), CD34(−), SMA(+), Des(−), Ki-67(+,60%)
Somatic mutation	BAP1(p.Glu200fs), PIK3CA(p.Glu545Lys), PTEN(p.Arg130*), TP53(p.Arg273His)	NA

### PDX model confirmation by IHC

**Fig. 3:**
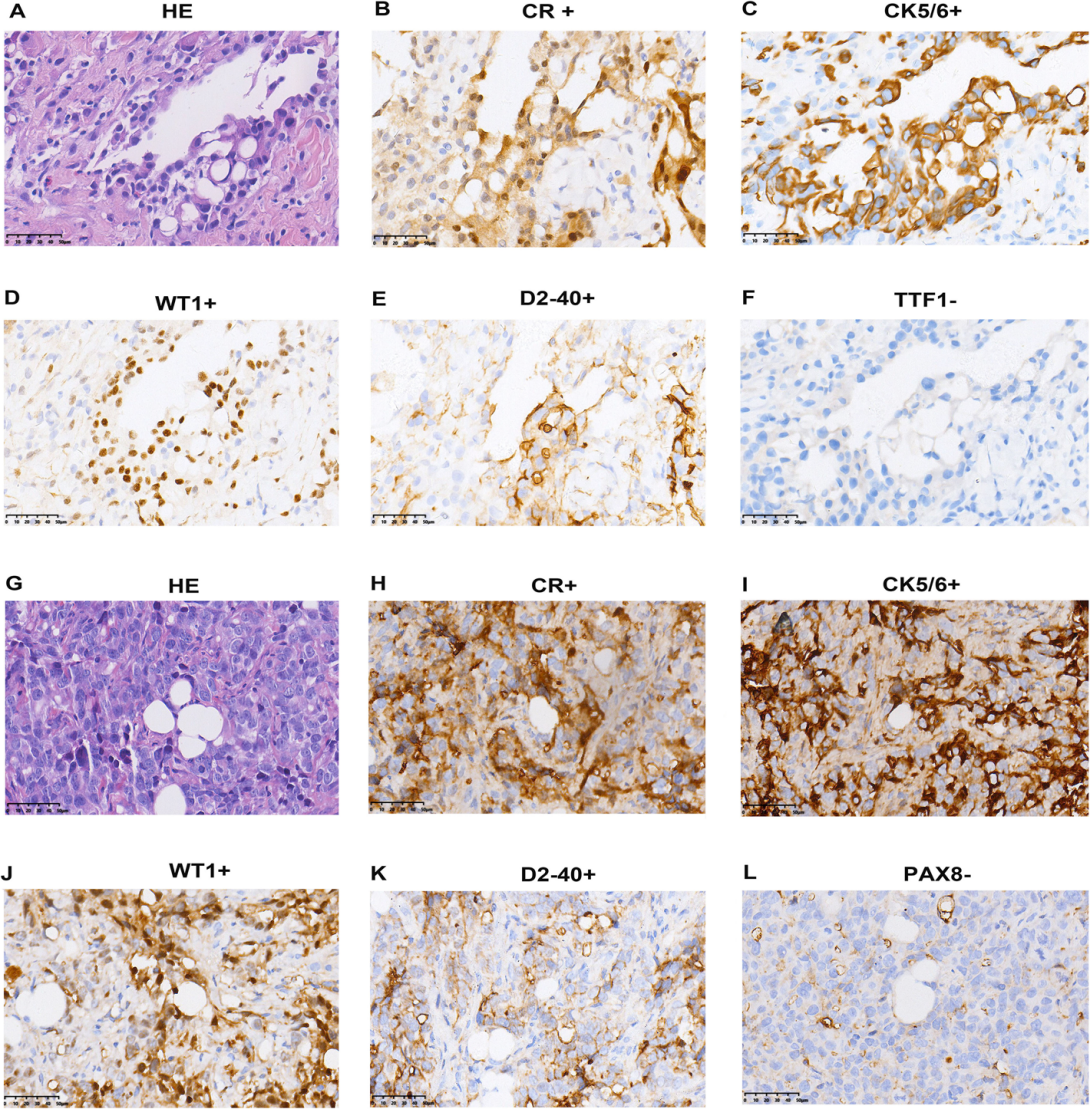
HE staining and IHC staining of tumor tissue from the original patient and PDX1 model (P3). For original tumor: (**A**) HE staining, (**B**-**E**) CR, CK5/6, WT1, and D2-40 positive (**F**) TTF1 negative; for PDX1’s tumor: (**G**) HE staining, (H-K) CR, CK5/6, WT1, and D2-40 positive, (**L**) PAX8 negative (original magnification ×400)

**Fig. 4:**
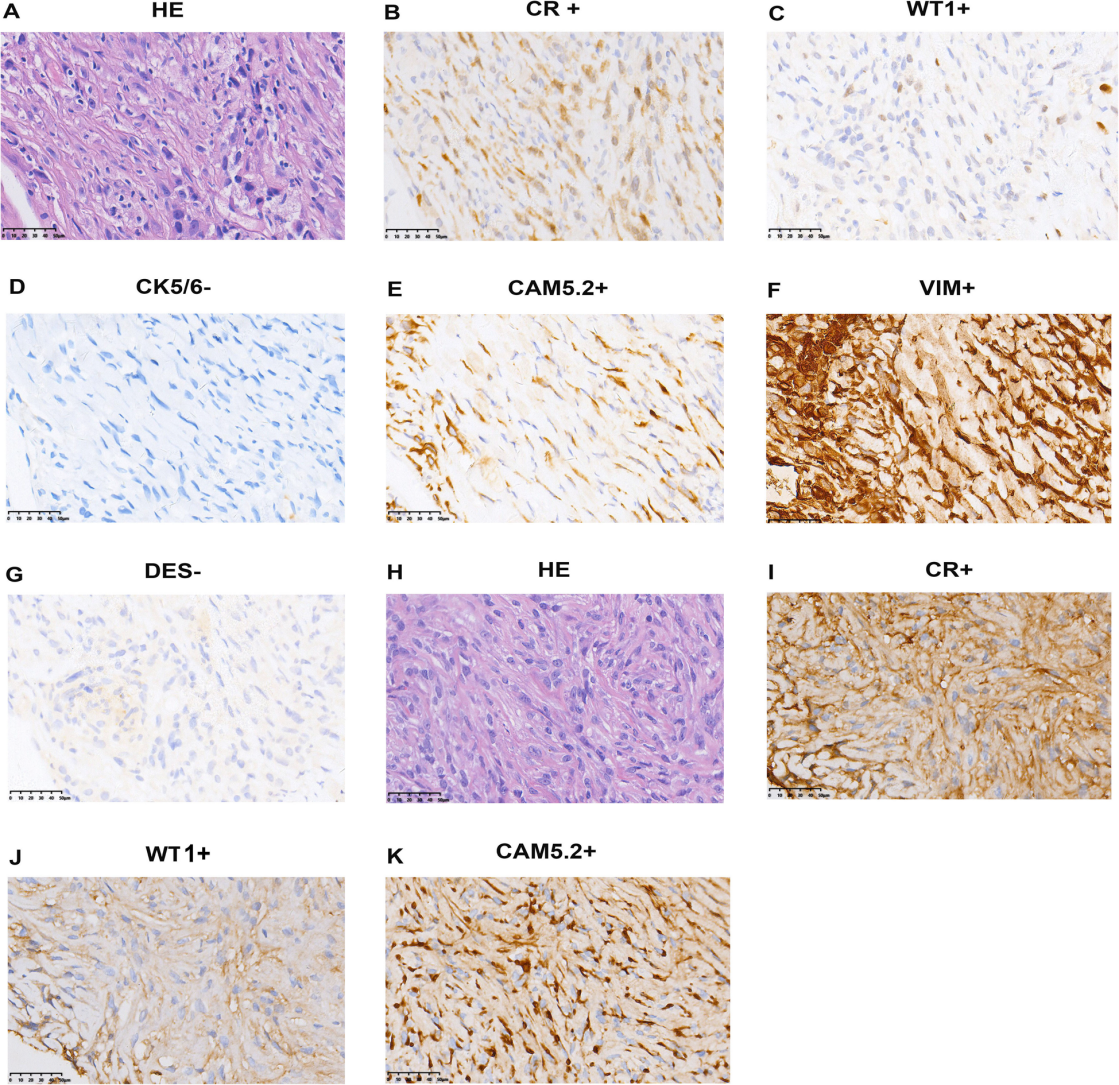
HE staining and IHC staining of tumor tissue from the original patient and PDX2 model (P3). For original tumor: (**A**) HE staining, (**B**, **C**, **E**, **F**) CR, WT1, CAM5.2, and VIM positive, (**D**, **G**) CK5/6 and DES negative; for PDX1’s tumor: (**H**) HE staining, (**I**-**K**) CR, WT1, CK5/6, CAM5.2 positive (original magnification ×400)

In the metabolomics study, 6 out of 10 PDX1 models (P3) and 8 out of 10 PDX2 (P3) model grew and were used for GC-MS based metabolomics. Figures 3 and 4 demonstrated Haemotoxylin and Eosin stains (HE) and IHC results of the two primary tumors and the xenograft tumors, PDX1 (Fig. 3) and PDX2 (Fig. 4). HE stains presented the epithelioid (Fig. 3A, G) and sarcomatoid (Fig. 4A, H) features of MPM for the primary tumors and PDX1&2 tumors. Positive expression of CR, CK5/6, WT1, and D2-40 (Fig. 3B-E) were denoted in primary epithelioid tumor tissue, and PDX1 expressed the same pattern (Fig. 3H-K). TTF1 negative in primary tumor (Fig. 3F) and PAX8 negative in PDX1 (Fig. 3L) distinguish MPM from metastatic lung adenocarcinomas. Altogether, these results confirmed the pathological subtype of epithelioid mesothelioma, and suggested successful construction of a reliable PDX model.

Similarly, expression of CR (Fig. 4B), and WT1 (Fig. 4C) confirmed the nature of the second model tumor being mesothelioma. CAM5.2 positive (Fig. 4E) distinguished this tumor from sarcoma. But CK5/6 (Fig. 4D) negative and VIM positive (Fig. 4F) can still discriminate its sarcomatoid identity from the epithelioid mesothelioma. In accordance, immunoprofiling for PDX2 showed CR (Fig. 4I), WT1 (Fig. 4J), and CAM5.2 (Fig. 4K) positive. In addition, DES (Fig. 4G) negative of primary tumor of PDX2 indicated poor differentiation ability of this tumor.

### Serum metabolic shift between PDX models and controls

In total, 209 metabolites in serum samples were annotated. After multivariate analyses, samples in PDX1 group were successfully separated from control group by unsupervised PCA (Fig. S1A), whereas PDX2 group cannot be separated from control group (Fig. S1B). In supervised PLS-DA models, clearer separation trend for PDX 1 (Fig. S1C) and 2 (Fig. S1D) were present, though the latter remains ambiguous. Volcano plots revealed significant changes (VIP > 1.0, *P*-value < 0.05) in metabolites in between PDX1 and 2 (Fig. S2), and each in comparison to controls (Fig. S1E, F). In PDX1 versus controls, 58 upregulated metabolites and 23 downregulated metabolites were obtained. And in PDX2, 21 upregulated metabolites and 3 downregulated metabolites were obtained. In addition, of the 12 overlapped metabolites, 10 were upregulated and 2 were downregulated, in both PDX1 and PDX2 versus controls. Between PDX1 and PDX2, 10 upregulated and 14 downregulated metabolites were presented. Detailed information of differential metabolites (including VIP, *P*-value, and fold change) was listed in Table S3 (PDX1 vs. control), Table S4 (PDX2 vs. control), Table S5 (overlapped in PDX1 and PDX2 compared to controls), and Table S6 (PDX1 vs. PDX2).

### Metabolic patterns and pathway enrichment

**Fig. 5:**
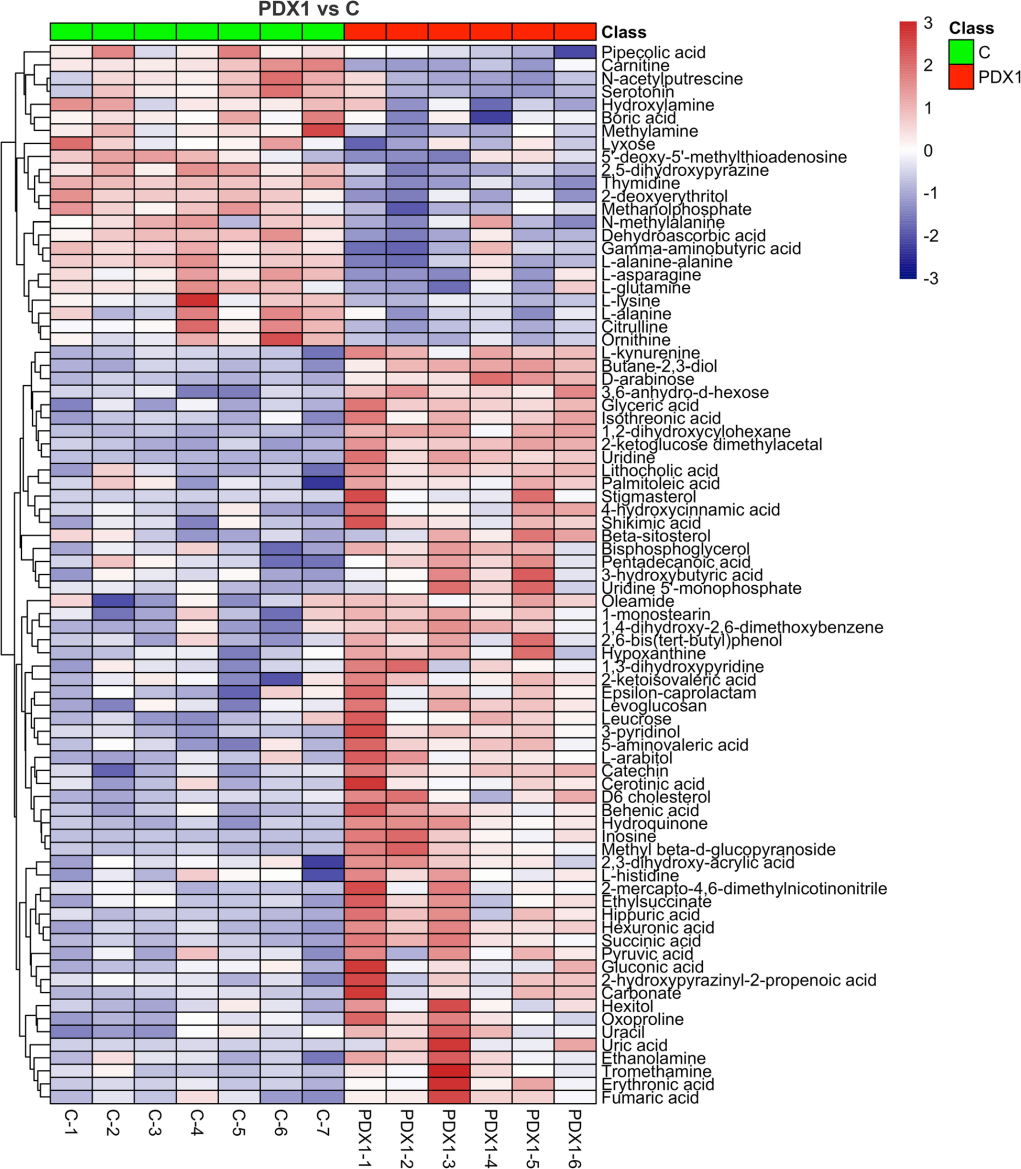
Heatmap plotting from serum-based metabolomics revealing the metabolic shift between PDX1 model and control. Differential metabolites with VIP > 1.0 and *P*-value < 0.05 were used in plotting

**Fig. 6:**
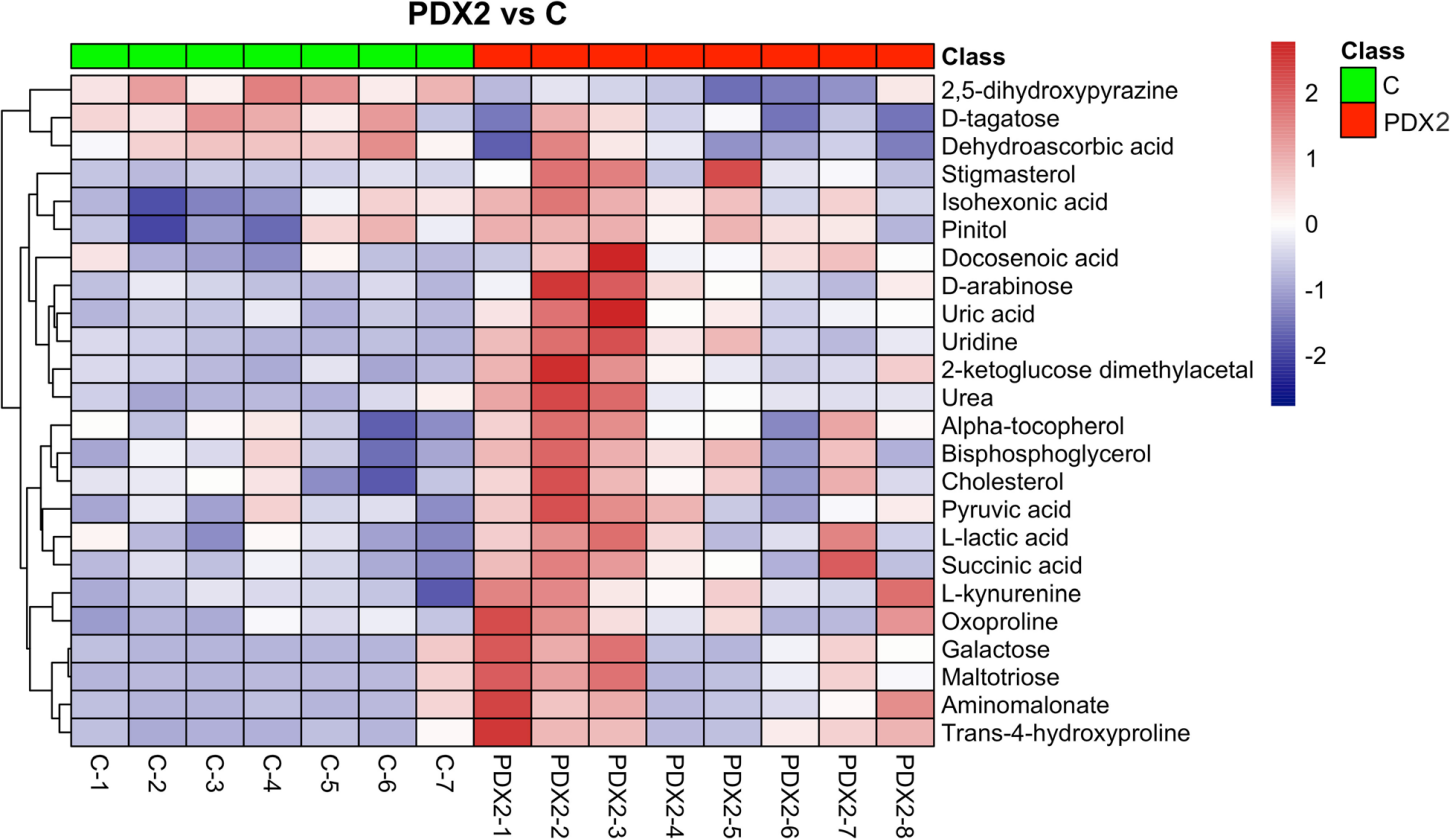
Heatmap plot for comparison between PDX2 and control. Differential metabolites with VIP > 1.0 and *P*-value < 0.05 were used in plotting

**Fig. 7:**
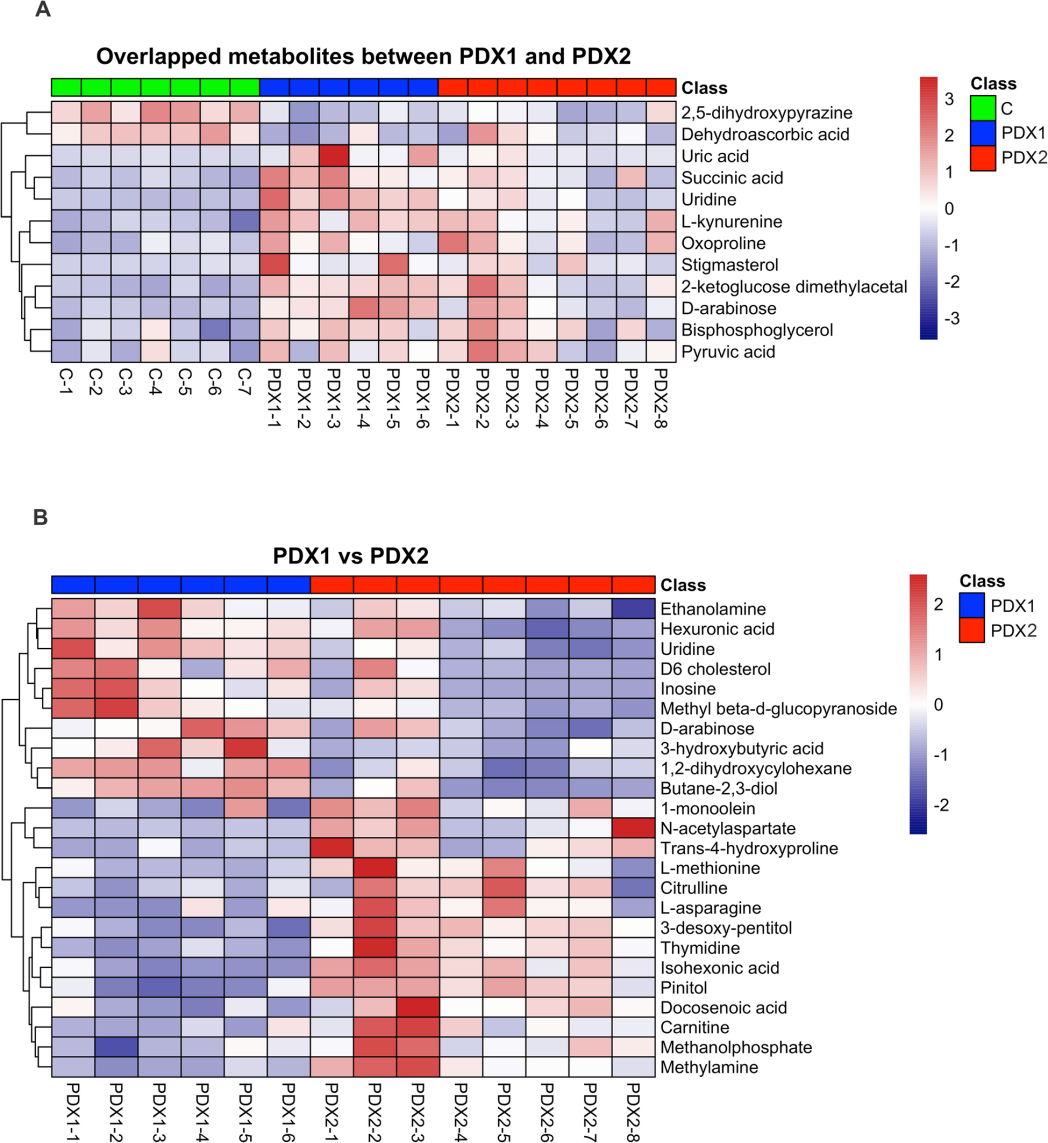
Heatmap plots for overlapped differential metabolites from PDX1 and PDX2 (A), and comparison between PDX1 and PDX2 (B). Differential metabolites with VIP > 1.0 and *P*-value < 0.05 were used in plotting

**Fig. 8:**
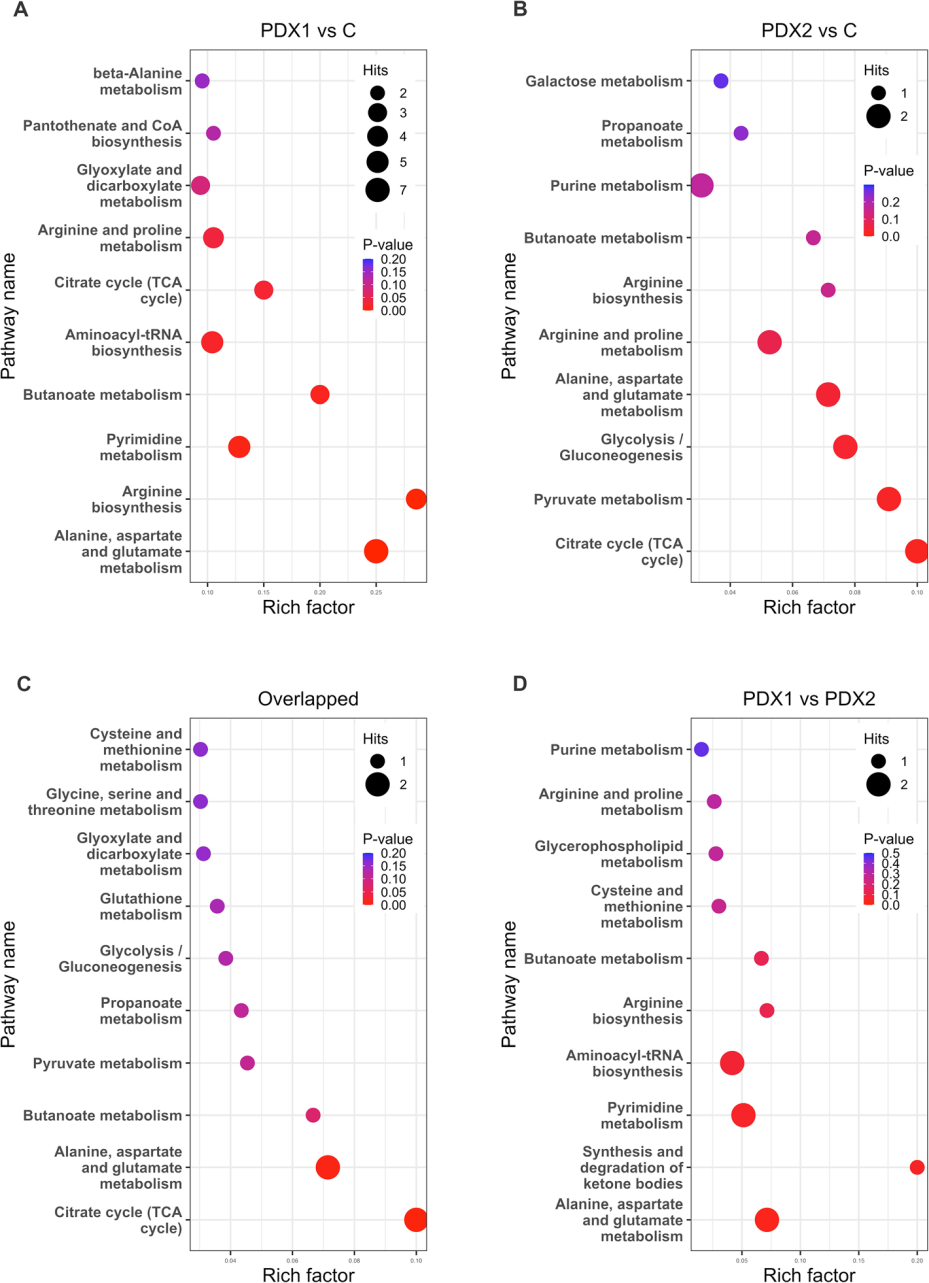
Pathway enrichment results for differential metabolites from PDX1 vs. control (A), PDX2 vs. control (B), overlapped differential metabolites between PDX1 and PDX2 (C), PDX1 vs. PDX2 (D)

**Fig. 9:**
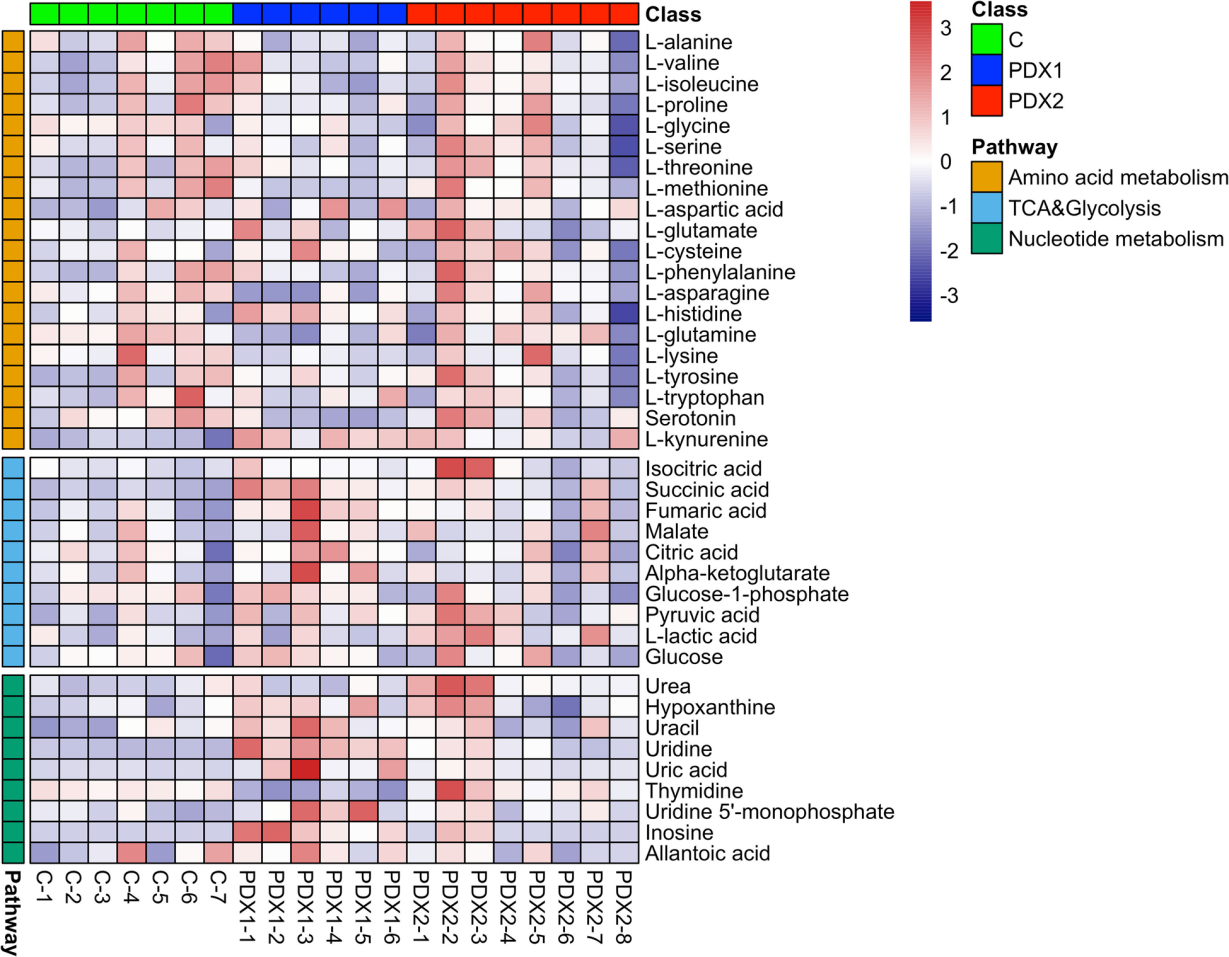
Heatmap plot for detected metabolites in amino acid metabolism, TCA & glycolysis, and nucleotide metabolism among PDX1, PDX2, and control

**Fig. 10:**
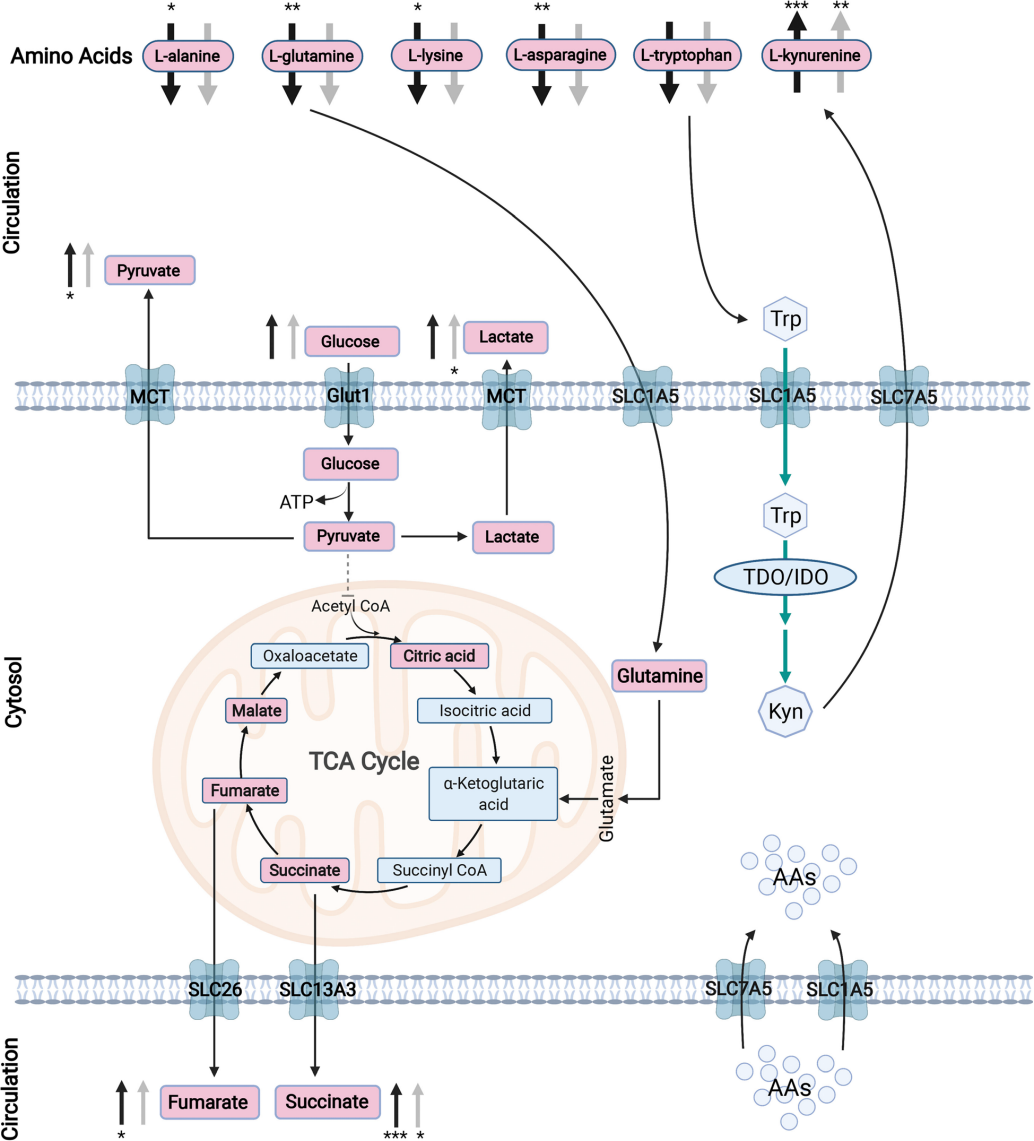
Illustration on the circulation of essential substances in and out of cancer cell. Glucose enters cancer cell through Glut1, undergoing glycolysis, forming pyruvate. Pyruvate does not go into mitochondria to synthesize Acetyl CoA, instead, it is directly exported out of cell or becomes lactate and excreted out of cell by membrane transporter MCT. Extracellular glutamines serve as fuels for the citric acid cycle. The intermediates succinate and fumarate are transported out of cell through transporters SLC13A3 and SLC26 respectively, causing the upregulation of these two metabolites in PDX serum. Amino acids get into cell through transporters SLC7A5 and SLC1A5. Tryptophan specifically, flow into cell through SLC1A5, degrade into kynurenine with the help of enzymes TDO and IDO. Kynurenine is then transported out of cell through SLC7A5. Solid black arrow denotes up or down regulation of differential metabolites found in PDX1 vs control, whereas solid gray arrow denotes the differential metabolites found in PDX2 vs control. *: *P*-value < 0.05; **: *P*-value < 0.01; ***: *P*-value < 0.001. Abbreviations: MCT, Monocarboxylate Transporter; Glut1, Glucose Transporter 1; SLC1A5, Solute Carrier Family 1 Member 5; SLC7A5, Solute Carrier Family 7 Member 5; SLC26, solute carrier 26; SLC13A3, Solute carrier Family 7 Member 5; TCA Cycle, Citric Acid Cycle; AAs, Amino Acids; Trp, Tryptophan; Kyn, Kynurenine

Hierarchical clustering heatmaps were then conducted and showed distinctive differentially expressed metabolic patterns between PDX1 and control (*n* = 82; Fig. 5), PDX2 and control (*n* = 25; Fig. 6), overlapped between PDX1 and 2 (*n* = 12; Fig. 7A), and between PDX1 and PDX2 (n = 25; Fig. 7B). Figure 8 illustrated enriched pathways of the differential metabolites between PDX1 vs. control (Fig. 8A, Table S7), PDX2 vs. control (Fig. 8B, Table S8), overlapped in PDX1 and PDX2 (Fig. 8C_,_ Table S9), and PDX1 vs. PDX2 (Fig. 8D, Table S10). Figure 9 showed all annotated metabolites presented and their enrichments in amino acid metabolism, TCA cycle and glycolysis, and nucleotide metabolism in PDX1, PDX2, and control. With the knowledge of the enriched pathways and the metabolites involved, an overall illustration of amino acid metabolism, tryptophan degradation, glycolysis, and TCA cycle was pictured in Fig. 10.

### Dysregulated amino acid metabolism in MPM

Among the 81 significant dysregulated metabolites presented in PDX1, 5 were amino acids with 4 downregulated and 1 upregulated. Within the 5 amino acids, upregulated kynurenine and downregulated serotonin were involved in tryptophan degradation. Meanwhile, 4 of top 10 most significantly enriched pathways in PDX1 were amino acid metabolism pathways, and according to rich factor and *P*-value, the aspartate and glutamine metabolism; and arginine biosynthesis were the top 2 enriched pathways.

In PDX2, no significant changes were observed in amino acids. Nevertheless, similar to PDX1, kynurenine was also significantly upregulated, with a fold change of 1.99 compared to controls. However, tryptophan itself did not display significant change in circulating levels in both PDX models.

### Dysregulated glycolysis and TCA metabolites in MPM

The top 3 most significantly enriched pathways in PDX2 were citrate cycle, pyruvate metabolism, and glycolysis/gluconeogenesis. In line, two metabolites in pyruvate metabolism, bisphosphoglycerol and pyruvic acid, were all upregulated, with fold changes of 1.46 and 1.37, respectively. Additionally, the end product of Warburg aerobic glycolysis, lactic acid, was found upregulated in PDX2 but not in PDX1.

Succinic acid and pyruvic acid are overlapped metabolites of TCA cycle in both PDX models that showed significant changes. Succinic acid  increased 2.17 folds in PDX1 and 1.49 folds in PDX2; and pyruvic acid increased 1.40 folds in PDX1 and 1.37 folds in PDX2. In addition, in PDX1, glutamine was downregulated to 0.49 folds, whereas fumaric acid was upregulated to 1.82 folds. But no significant alterations in other TCA compartments were detected in PDX2.

### Abnormality in nucleotide metabolism

We detected uridine 5′-monophosphate (UMP), uridine, thymidine, and uracil enrichment in pyrimidine metabolism. In addition, hypoxanthine, inosine, allantoate, uric acid, and urea were enriched in purine metabolism. Consistently, uric acid, uridine, and inosine were significantly upregulated in both PDX1 and PDX2. Uracil, UMP, and hypoxanthine were upregulated in both models, whereas the trends were only significant in PDX1. Urea was only significantly increased in PDX2 with a fold change of 1.40, while thymidine was only downregulated in PDX1 with a fold change of 0.40. No change was detected in levels of allantoate in both models.

## Discussion

Sample scarcity has been an issue for research in MPM. Our methodology for PDX modeling from US-guided pleural biopsy in part removes this restraint, as biopsy samples are more accessible than samples from surgery. Although biopsy during video-assisted thoracoscopic surgery (VATS) was the gold standard for MPM diagnosis in current, it is more invasive than US-guided biopsy. Notably, dedicated examination and sampling used in our strategy helped improve efficiency as well as reduce the cost in modeling. For pleural lesions, a US-guided pleural biopsy was utilized, which outstands for its real-time multiplanar visualization that aids sampling [18]. Then the senior sonographer helped narrow specimens down to MPM candidates for modeling through the aforementioned exclusion criteria. Precise diagnosis by an experienced pathologist then helped rule out non-MPM PDXs. Thereby, our modeling strategy can diminish the waste of resources and time while retaining the reliability of PDX models.

In addition, PDX models offer a more manipulative environment for developing therapies and detecting biomarkers compared to human beings [19], as well as better retain heterogeneity of tumor compared to cell lines [8]. Many cancer studies use PDX model as it highly resembles the primary tumor implanted into it [20–23]. Meanwhile, such model parallels the original tumor better than cell lines, and concurrent results are closely relevant to the clinic [8]. Hence, for the above reasons, this modeling strategy is insightful and feasible, which can facilitate not only MPM-related research but also the research of other rare cancers limited by lack of specimens.

By further applying metabolomics based on PDX sera, we detected a panel of dysregulated metabolites and enriched pathways. A significant proportion of metabolic changes denoted in our results was verified in other clinical studies, indicating the reliability of both our metabolomics method and the PDX model. Therefore, we encourage the use of US-guided pleural biopsy for PDX modeling in combination with metabolomics for investigation in rare cancers, including MPM, which brings an opportunity for the identification of predictive markers and treatment targets.

Our metabolomics results revealed a dysregulated amino acid metabolism in MPM. Many studies had reported that cancers vastly demand amino acid for ATP yielding, growth, and progression, through overexpression of SLC7A5 and SLC1A5 [24–26]. Our results suggested MPM has same desire as other cancers for amino acids. Particularly, serum kynurenine, which is a downstream metabolite of tryptophan, increased significantly in both PDX1 and PDX2 in comparison to controls, suggesting an increased tryptophan metabolism. Some cancer research had opined immunosuppressive role of kynurenine [27]. In line, many studies documented increased levels of IDO and TDO in cancers, which are two enzymes catalyzing anabolism of tryptophan to kynurenine [28–30]. Collectively, dysregulated circulating amino acid metabolism, especially kynurenine metabolism, was a significant metabolic feature of MPM.

We also revealed dysregulations in TCA cycle and glycolysis in MPM. We detected an increase in pyruvic acid efflux, suggesting an elevated glycolysis rate. However, contradictory results were found in lactic acid, which was elevated in PDX2 but unchanged in PDX1, denoting a higher rate of aerobic glycolysis in PDX2 than PDX1. Increasing evidence has suggested that lactic acid secretion helps immune evasion of tumor by constructing a micro-environment with low pH that suppresses anti-tumor immune response [31]. Therefore, the increased glycolysis rate in MPM may have dual purposes that increase energy fueling as well as help tumor escape from immunity.

Dysregulations in metabolites of purine metabolism and pyrimidine metabolism were detected, indicating an imbalanced nucleotide metabolism. Based on our results, circulating uridine was significantly upregulated in both PDX models, which may be signs of a higher rate of uridine synthesis in MPM. Tumor synthesizes more nucleotides, increasing deoxyribonucleic acid (DNA) and ribonucleic acid (RNA) pools that support proliferation [32]. Further we found significant elevations in uric acid, which has been reported to be released from dying tumor cells [33]. This also frequently occurs in other cancers, such as breast cancer [34], hepatocarcinoma [35], and head and neck carcinoma [36]. Uric acid also promotes tumor immune rejection [33], and serves as a pro-oxidant that induces tumor growth [37]. Herein, the excessively synthesized uridine and uric acid in MPM may be biomarkers for MPM.

However, the changed metabolites were only phenotypes which lack clear molecular mechanisms to be elucidated. Also, it is well-known that BAP1 influences metabolism in MPM, so metabolic inconsistency between samples of different BAP1 status should be compared. It is a pity that we only have one patient’s information of genetic mutation, but future research with larger sample size and complete clinical information should fill in this gap.

## Conclusions

In conclusion, our study developed a novel modeling technique to facilitate research in malignant mesothelioma, especially in China. In using a combination of CT scanning, pathological analysis and US-guided pleural biopsy for PDX modeling, we can remove barriers in MPM research that is caused by the scarcity in samples, thus improving availability of research in MPM or other infrequent cancers. By further coupling with metabolomics to screen for metabolic biomarkers, we can advance the current diagnostic method and treatment for MM. Nevertheless, studies with larger sample size are needed for this cancer, and molecular mechanisms as well as genetic predispositions should be further investigated to verify our results.

## Supplementary Information

**Additional file 1.**

**Additional file 2.**

**Additional file 3.**

**Additional file 4.**

## Data Availability

All data generated or analyzed during this study are included in this published article and its supplementary information files.

## Abbreviations

MM: Malignant mesothelioma
MPM: Malignant pleural mesothelioma
PDX: Patient-derived xenograft
US: Ultrasound
CT: Computed tomography
IHC: Immunohistochemistry
GC-MS: Gas chromatography-mass spectrometry
PCA: Principal component analysis
PLS-DA: Partial least-squares-discriminant analysis
HE: Hematoxylin and Eosin
UMP: Uridine 5′-monophosphate
DNA: Deoxyribonucleic acid
RNA: Ribonucleic acid
TCA: Tricarboxylic acid
VIP: Variable importance in the projection
VATS: Video-assisted thoracoscopic surgery
