# Supplementary material for: A novel PDX modeling strategy and its application in metabolomics study for malignant pleural mesothelioma
Source: BMC Cancer. 2021 Nov 17;21:1235. doi: 10.1186/s12885-021-08980-5 (PMC8600931; doi:10.1186/s12885-021-08980-5)
Supplement: Supplementary file 1 — Additional file 1. [file 12885_2021_8980_MOESM1_ESM.docx]

**Table S1**: **Summary of 116 patients in ultrasound-guided pleural biopsy**

| **Pathological diagnosis** | **Number of cases** | **Percentage (%)** |
| --- | --- | --- |
| Malignant pleural mesothelioma | 7 | 6.0 |
| Lung cancer pleural metastasis | 57 | 49.1 |
| Non-lung cancer pleural metastasis | 23 | 19.8 |
| Rhabdomyosarcoma | 2 | 1.7 |
| Tuberculosis | 10 | 8.6 |
| Non-specific benign lesions | 9 | 7.8 |
| Schwannoma | 3 | 2.6 |
| Unclear diagnosis | 5 | 4.3 |
| Total | 116 | 100 |

**Table S2**: **Summary of 14 patients for PDX model candidates**

| **Case** | **Pathological**  **diagnosis** | **Feature in**  **CT imaging** | **Age**  **(year)** | **Sex** | **Remark** |
| --- | --- | --- | --- | --- | --- |
| 1 | malignant pleural mesothelioma | Pleural thickening of annular nodules, lumps in the lungs and chest wall | 55 | Male | Succeed |
| 2 | malignant pleural mesothelioma | Pleural thickening of annular nodules, chest wall lumps | 85 | Female | Succeed |
| 3 | malignant pleural mesothelioma | Pleural annular nodular thickening | 47 | Male | Failed |
| 4 | malignant pleural mesothelioma | Pleural annular nodular thickening | 53 | Female | Failed |
| 5 | malignant pleural mesothelioma | Pleural annular nodular thickening | 55 | Female | Failed |
| 6 | malignant pleural mesothelioma | Pleural annular nodular thickening | 66 | Female | Excluded due to previous treatment |
| 7 | malignant pleural mesothelioma | Pleural annular nodular thickening | 40 | Female | Excluded due to previous treatment |
| 8 | Lung adenocarcinoma | Massive thickening of pleural ring nodules, masses in the lungs | 55 | Male | Excluded after diagnosis |
| 9 | Lung adenocarcinoma | Massive thickening of pleural ring nodules, masses in the lungs | 62 | Male | Excluded after diagnosis |
| 10 | Lung adenocarcinoma | Massive thickening of pleural ring nodules, masses in the lungs | 60 | Female | Excluded after diagnosis |
| 11 | Rhabdomyosarcoma | Lump-like thickening of the pleura | 16 | Male | Excluded after diagnosis |
| 12 | Rhabdomyosarcoma | Lump-like thickening of the pleura | 18 | Male | Excluded after diagnosis |
| 13 | Spindle cell malignancies | Lump-like thickening of the pleura | 78 | Male | Inadequate specimen, only for diagnosis |
| 14 | Poorly differentiated cancer, not sure | Massive thickening of pleural ring nodules | 65 | Male | Refuse to participate |

**Table S3: Differential metabolites between PDX1 model and control group**

| **No.** | **Metabolite** | **VIP^a^** | **P-value^b^** | **FC^c^** | **No.** | **Metabolite** | **VIP^a^** | **P-value^b^** | **FC^c^** |
| --- | --- | --- | --- | --- | --- | --- | --- | --- | --- |
| 1 | 2-ketoglucose dimethylacetal | 1.77 | 2.7E-07 | 1.74 | 42 | L-arabitol | 1.24 | 1.1E-02 | 1.23 |
| 2 | Thymidine | 1.77 | 3.7E-07 | 0.40 | 43 | Oxoproline | 1.26 | 1.1E-02 | 1.51 |
| 3 | Butane-2,3-diol | 1.78 | 9.3E-07 | 2.20 | 44 | Hypoxanthine | 1.25 | 1.1E-02 | 1.23 |
| 4 | 1,2-dihydroxycylohexane | 1.73 | 2.2E-06 | 2.15 | 45 | Fumaric acid | 1.24 | 1.1E-02 | 1.82 |
| 5 | Uridine | 1.71 | 3.1E-06 | 6.60 | 46 | 4-hydroxycinnamic acid | 1.24 | 1.2E-02 | 1.18 |
| 6 | Hexuronic acid | 1.66 | 2.4E-05 | 1.71 | 47 | Carbonate | 1.24 | 1.3E-02 | 1.25 |
| 7 | 2,5-dihydroxypyrazine | 1.68 | 3.9E-05 | 0.55 | 48 | Ethanolamine | 1.25 | 1.4E-02 | 1.40 |
| 8 | D-arabinose | 1.72 | 4.5E-05 | 6.14 | 49 | Ethylsuccinate | 1.29 | 1.4E-02 | 1.14 |
| 9 | 2-deoxyerythritol | 1.66 | 5.6E-05 | 0.82 | 50 | 2,6-bis(tert-butyl)phenol | 1.22 | 1.4E-02 | 1.09 |
| 10 | Glyceric acid | 1.62 | 5.9E-05 | 1.44 | 51 | Erythronic acid | 1.20 | 1.5E-02 | 1.54 |
| 11 | L-kynurenine | 1.61 | 9.6E-05 | 2.99 | 52 | Hydroxylamine | 1.36 | 1.6E-02 | 0.81 |
| 12 | Hydroquinone | 1.58 | 1.5E-04 | 1.27 | 53 | Pipecolic acid | 1.28 | 1.6E-02 | 0.84 |
| 13 | Carnitine | 1.58 | 1.5E-04 | 0.53 | 54 | 2-ketoisovaleric acid | 1.19 | 1.7E-02 | 1.11 |
| 14 | Dehydroascorbic acid | 1.58 | 1.6E-04 | 0.56 | 55 | 1-monostearin | 1.17 | 1.9E-02 | 1.18 |
| 15 | Isothreonic acid | 1.58 | 1.6E-04 | 1.45 | 56 | Uracil | 1.18 | 1.9E-02 | 1.41 |
| 16 | Catechin | 1.59 | 1.9E-04 | 1.33 | 57 | 2-mercapto-4,6-dimethylnicotinonitrile | 1.24 | 2.2E-02 | 1.06 |
| 17 | 3,6-anhydro-d-hexose | 1.60 | 2.1E-04 | 1.45 | 58 | 3-hydroxybutyric acid | 1.14 | 2.3E-02 | 2.18 |
| 18 | L-alanine-alanine | 1.54 | 3.5E-04 | 0.78 | 59 | Hexitol | 1.15 | 2.4E-02 | 1.34 |
| 19 | Succinic acid | 1.54 | 3.9E-04 | 2.17 | 60 | Cerotinic acid | 1.14 | 2.7E-02 | 1.65 |
| 20 | Lithocholic acid | 1.50 | 6.0E-04 | 1.73 | 61 | 1,3-dihydroxypyridine | 1.12 | 2.7E-02 | 1.13 |
| 21 | Inosine | 1.49 | 7.7E-04 | 57.24 | 62 | L-lysine | 1.12 | 2.8E-02 | 0.63 |
| 22 | 3-pyridinol | 1.49 | 8.4E-04 | 1.20 | 63 | Palmitoleic acid | 1.14 | 2.8E-02 | 1.58 |
| 23 | Methyl beta-d-glucopyranoside | 1.47 | 1.0E-03 | 3.81 | 64 | Pentadecanoic acid | 1.12 | 2.8E-02 | 1.34 |
| 24 | Methanolphosphate | 1.48 | 1.1E-03 | 0.68 | 65 | Boric acid | 1.19 | 2.8E-02 | 0.84 |
| 25 | Hippuric acid | 1.47 | 1.3E-03 | 1.36 | 66 | Uric acid | 1.12 | 2.8E-02 | 5.20 |
| 26 | 1,4-dihydroxy-2,6-dimethoxybenzene | 1.45 | 1.6E-03 | 1.51 | 67 | 2,3-dihydroxy-acrylic acid | 1.13 | 3.0E-02 | 1.35 |
| 27 | Behenic acid | 1.42 | 1.9E-03 | 1.44 | 68 | Oleamide | 1.17 | 3.0E-02 | 1.12 |
| 28 | Citrulline | 1.41 | 2.1E-03 | 0.47 | 69 | Lyxose | 1.10 | 3.0E-02 | 0.85 |
| 29 | Levoglucosan | 1.36 | 3.6E-03 | 1.10 | 70 | Uridine 5'-monophosphate | 1.10 | 3.2E-02 | 1.79 |
| 30 | Bisphosphoglycerol | 1.36 | 4.1E-03 | 1.60 | 71 | 2-hydroxypyrazinyl-2-propenoic acid | 1.16 | 3.2E-02 | 1.05 |
| 31 | Shikimic acid | 1.36 | 4.1E-03 | 1.16 | 72 | L-alanine | 1.08 | 3.5E-02 | 0.71 |
| 32 | 5-aminovaleric acid | 1.35 | 4.3E-03 | 1.14 | 73 | 5'-deoxy-5'-methylthioadenosine | 1.08 | 3.5E-02 | 0.84 |
| 33 | L-asparagine | 1.36 | 4.5E-03 | 0.35 | 74 | L-histidine | 1.10 | 3.6E-02 | 1.23 |
| 34 | Gamma-aminobutyric acid | 1.35 | 4.5E-03 | 0.62 | 75 | Stigmasterol | 1.08 | 3.8E-02 | 3.44 |
| 35 | L-glutamine | 1.32 | 5.8E-03 | 0.49 | 76 | Gluconic acid | 1.07 | 4.0E-02 | 1.58 |
| 36 | Serotonin | 1.36 | 6.0E-03 | 0.75 | 77 | Epsilon-caprolactam | 1.07 | 4.0E-02 | 1.10 |
| 37 | N-acetylputrescine | 1.35 | 6.0E-03 | 0.74 | 78 | Tromethamine | 1.06 | 4.1E-02 | 2.12 |
| 38 | Methylamine | 1.29 | 7.9E-03 | 0.79 | 79 | Pyruvic acid | 1.07 | 4.4E-02 | 1.40 |
| 39 | D6 cholesterol | 1.27 | 8.5E-03 | 1.92 | 80 | N-methylalanine | 1.04 | 4.4E-02 | 0.93 |
| 40 | Ornithine | 1.26 | 9.5E-03 | 0.45 | 81 | Beta-sitosterol | 1.08 | 4.6E-02 | 1.34 |
| 41 | Leucrose | 1.25 | 1.1E-02 | 1.42 |  |  |  |  |  |

^a^: Variable importance in the projection of PLS-DA model.

^b^: From two-tailed Student’s t-test test.

^c^: Fold change (PDX model vs control).

**Table S4: Differential metabolites between PDX2 model and control group**

| **No.** | **Metabolite** | **VIP^a^** | **P-value^b^** | **FC^c^** |
| --- | --- | --- | --- | --- |
| 1 | 2,5-dihydroxypyrazine | 2.46 | 1.8E-04 | 0.63 |
| 2 | L-kynurenine | 1.79 | 3.3E-03 | 1.99 |
| 3 | Uridine | 1.43 | 8.7E-03 | 2.69 |
| 4 | Trans-4-hydroxyproline | 1.54 | 1.0E-02 | 6.79 |
| 5 | Succinic acid | 1.40 | 1.3E-02 | 1.49 |
| 6 | 2-ketoglucose dimethylacetal | 1.35 | 1.4E-02 | 1.50 |
| 7 | Uric acid | 1.33 | 1.5E-02 | 1.97 |
| 8 | Isohexonic acid | 1.40 | 1.7E-02 | 1.94 |
| 9 | L-lactic acid | 1.34 | 1.8E-02 | 1.33 |
| 10 | Dehydroascorbic acid | 2.15 | 2.3E-02 | 0.72 |
| 11 | D-tagatose | 2.14 | 2.3E-02 | 0.65 |
| 12 | Oxoproline | 1.31 | 2.8E-02 | 1.50 |
| 13 | Urea | 1.22 | 2.9E-02 | 1.40 |
| 14 | Bisphosphoglycerol | 1.23 | 3.1E-02 | 1.46 |
| 15 | Docosenoic acid | 1.27 | 3.2E-02 | 1.92 |
| 16 | D-arabinose | 1.20 | 3.3E-02 | 2.98 |
| 17 | Galactose | 1.20 | 3.5E-02 | 35.23 |
| 18 | Pinitol | 1.36 | 3.6E-02 | 1.44 |
| 19 | Aminomalonate | 1.26 | 3.6E-02 | 5.87 |
| 20 | Maltotriose | 1.18 | 3.7E-02 | 7.43 |
| 21 | Stigmasterol | 1.19 | 4.0E-02 | 2.08 |
| 22 | Cholesterol | 1.15 | 4.4E-02 | 1.27 |
| 23 | Pyruvic acid | 1.13 | 4.6E-02 | 1.37 |
| 24 | Alpha-tocopherol | 1.12 | 4.8E-02 | 1.28 |

^a^: Variable importance in the projection of PLS-DA model.

^b^: From two-tailed Student’s t-test test.

^c^: Fold change (PDX model vs control).

**Table S5: Overlapped differential metabolites from PDX1 and PDX2 models**

| **No.** | **Metabolite** | **PDX1** | | |  | **PDX2** | | |
| --- | --- | --- | --- | --- | --- | --- | --- | --- |
|  |  | **VIP^a^** | **P-value^b^** | **FC^c^** |  | **VIP** | **P-value** | **FC** |
| 1 | 2-ketoglucose dimethylacetal | 1.77 | 2.7E-07 | 1.74 |  | 1.35 | 1.4E-02 | 1.50 |
| 2 | Uridine | 1.71 | 3.1E-06 | 6.60 |  | 1.43 | 8.7E-03 | 2.69 |
| 3 | 2,5-dihydroxypyrazine | 1.68 | 3.9E-05 | 0.55 |  | 2.46 | 1.8E-04 | 0.63 |
| 4 | D-arabinose | 1.72 | 4.5E-05 | 6.14 |  | 1.20 | 3.3E-02 | 2.98 |
| 5 | L-kynurenine | 1.61 | 9.6E-05 | 2.99 |  | 1.79 | 3.3E-03 | 1.99 |
| 6 | Dehydroascorbic acid | 1.58 | 1.6E-04 | 0.56 |  | 2.15 | 2.3E-02 | 0.72 |
| 7 | Succinic acid | 1.54 | 3.9E-04 | 2.17 |  | 1.40 | 1.3E-02 | 1.49 |
| 8 | Bisphosphoglycerol | 1.36 | 4.1E-03 | 1.60 |  | 1.23 | 3.1E-02 | 1.46 |
| 9 | Oxoproline | 1.26 | 1.1E-02 | 1.51 |  | 1.31 | 2.8E-02 | 1.50 |
| 10 | Uric acid | 1.12 | 2.8E-02 | 5.20 |  | 1.33 | 1.5E-02 | 1.97 |
| 11 | Stigmasterol | 1.08 | 3.8E-02 | 3.44 |  | 1.19 | 4.0E-02 | 2.08 |
| 12 | Pyruvic acid | 1.07 | 4.4E-02 | 1.40 |  | 1.13 | 4.6E-02 | 1.37 |

^a^: Variable importance in the projection of PLS-DA model.

^b^: From two-tailed Student’s t-test test.

^c^: Fold change (PDX vs control).

**Table S6: Differential metabolites between PDX1 and PDX2 models**

| **No.** | **Metabolite** | **VIP^a^** | **P-value^b^** | **FC^c^** | **No.** | **Metabolite** | **VIP** | **P-value** | **FC** |
| --- | --- | --- | --- | --- | --- | --- | --- | --- | --- |
| 1 | 1,2-dihydroxycylohexane | 2.38 | 1.6E-04 | 1.85 | 17 | Ethanolamine | 1.73 | 2.10E-02 | 1.41 |
| 2 | Pinitol | 2.36 | 1.7E-04 | 0.45 | 18 | Methanolphosphate | 1.71 | 2.20E-02 | 0.73 |
| 3 | Isohexonic acid | 2.33 | 2.6E-04 | 0.35 | 19 | Trans-4-hydroxyproline | 1.65 | 2.80E-02 | 0.31 |
| 4 | Butane-2,3-diol | 2.34 | 2.8E-04 | 1.9 | 20 | 1-monoolein | 1.61 | 3.20E-02 | 0.52 |
| 5 | 3-desoxy-pentitol | 2.28 | 4.5E-04 | 0.39 | 21 | N-acetylaspartate | 1.60 | 3.40E-02 | 0.06 |
| 6 | Uridine | 2.24 | 7.1E-04 | 2.4 | 22 | D-arabinose | 1.63 | 3.60E-02 | 1.9 |
| 7 | Thymidine | 2.14 | 1.7E-03 | 0.37 | 23 | L-asparagine | 1.55 | 4.20E-02 | 0.38 |
| 8 | Methylamine | 2.01 | 4.3E-03 | 0.67 | 24 | D6 cholesterol | 1.57 | 4.30E-02 | 1.78 |
| 9 | 3-hydroxybutyric acid | 2.00 | 4.3E-03 | 2.59 | 25 | Hexuronic acid | 1.56 | 4.40E-02 | 1.35 |
| 10 | Methyl beta-d-glucopyranoside | 1.97 | 5.5E-03 | 2.37 | 26 | L-methionine | 1.54 | 4.50E-02 | 0.49 |
| 11 | Docosenoic acid | 1.82 | 1.2E-02 | 0.35 | 27 | Citrulline | 1.53 | 4.50E-02 | 0.59 |
| 12 | Inosine | 1.83 | 1.3E-02 | 4.1 | 28 | Carnitine | 1.53 | 4.70E-02 | 0.68 |

^a^: Variable importance in the projection of PLS-DA model.

^b^: From two-tailed Student’s t-test test.

^c^: Fold change (PDX1 vs PDX2).

**Table S7: Pathways enriched of differential metabolites in PDX1**

| **Pathway** | **Total^a^** | **Hits^b^** | **P-value** | **-log_10_P** | | **Rich factor^c^** | |
| --- | --- | --- | --- | --- | --- | --- | --- |
| Alanine, aspartate and glutamate metabolism | 28 | 7 | 1.92E-05 | 4.718 | 0.250 | |  |
| Arginine biosynthesis | 14 | 4 | 8.17E-04 | 3.088 | 0.286 | |  |
| Pyrimidine metabolism | 39 | 5 | 7.80E-03 | 2.108 | 0.128 | |  |
| Butanoate metabolism | 15 | 3 | 1.16E-02 | 1.937 | 0.200 | |  |
| Aminoacyl-tRNA biosynthesis | 48 | 5 | 1.85E-02 | 1.732 | 0.104 | |  |
| Citrate cycle (TCA cycle) | 20 | 3 | 2.58E-02 | 1.589 | 0.150 | |  |
| Arginine and proline metabolism | 38 | 4 | 3.36E-02 | 1.474 | 0.105 | |  |
| Glyoxylate and dicarboxylate metabolism | 32 | 3 | 8.52E-02 | 1.070 | 0.094 | |  |
| Pantothenate and CoA biosynthesis | 19 | 2 | 1.27E-01 | 0.895 | 0.105 | |  |
| beta-Alanine metabolism | 21 | 2 | 1.50E-01 | 0.824 | 0.095 | |  |
| Synthesis and degradation of ketone bodies | 5 | 1 | 1.54E-01 | 0.812 | 0.200 | |  |
| Pentose phosphate pathway | 22 | 2 | 1.62E-01 | 0.791 | 0.091 | |  |
| Pyruvate metabolism | 22 | 2 | 1.62E-01 | 0.791 | 0.091 | |  |
| Purine metabolism | 65 | 4 | 1.62E-01 | 0.790 | 0.062 | |  |
| D-Glutamine and D-glutamate metabolism | 6 | 1 | 1.82E-01 | 0.740 | 0.167 | |  |
| Nitrogen metabolism | 6 | 1 | 1.82E-01 | 0.740 | 0.167 | |  |
| Lysine degradation | 25 | 2 | 1.98E-01 | 0.704 | 0.080 | |  |
| Glutathione metabolism | 28 | 2 | 2.34E-01 | 0.630 | 0.071 | |  |
| Valine, leucine and isoleucine biosynthesis | 8 | 1 | 2.35E-01 | 0.628 | 0.125 | |  |
| Phenylalanine metabolism | 10 | 1 | 2.85E-01 | 0.545 | 0.100 | |  |
| Biotin metabolism | 10 | 1 | 2.85E-01 | 0.545 | 0.100 | |  |
| Cysteine and methionine metabolism | 33 | 2 | 2.96E-01 | 0.528 | 0.061 | |  |
| Glycine, serine and threonine metabolism | 33 | 2 | 2.96E-01 | 0.528 | 0.061 | |  |
| Tryptophan metabolism | 41 | 2 | 3.94E-01 | 0.405 | 0.049 | |  |
| Tyrosine metabolism | 42 | 2 | 4.06E-01 | 0.392 | 0.048 | |  |
| Glycerolipid metabolism | 16 | 1 | 4.16E-01 | 0.381 | 0.063 | |  |
| Histidine metabolism | 16 | 1 | 4.16E-01 | 0.381 | 0.063 | |  |
| Pentose and glucuronate interconversions | 18 | 1 | 4.54E-01 | 0.343 | 0.056 | |  |
| Selenocompound metabolism | 20 | 1 | 4.90E-01 | 0.310 | 0.050 | |  |
| Propanoate metabolism | 23 | 1 | 5.39E-01 | 0.268 | 0.043 | |  |
| Glycolysis / Gluconeogenesis | 26 | 1 | 5.84E-01 | 0.234 | 0.038 | |  |
| Galactose metabolism | 27 | 1 | 5.98E-01 | 0.223 | 0.037 | |  |
| Glycerophospholipid metabolism | 36 | 1 | 7.04E-01 | 0.152 | 0.028 | |  |
| Valine, leucine and isoleucine degradation | 40 | 1 | 7.42E-01 | 0.129 | 0.025 | |  |
| Steroid biosynthesis | 42 | 1 | 7.59E-01 | 0.120 | 0.024 | |  |

^a^: Number of total metabolites in pathway; ^b^: Number of metabolites detected; ^c^: ratio of detected metabolites

in the pathway. Result from online tool (https://www.metaboanalyst.ca).

**Table S8: Pathway enriched of differential metabolites in PDX2**

| **Pathway** | **Total^a^** | **Hits^b^** | **P-value** | **-log_10_P** | **Rich factor^c^** |
| --- | --- | --- | --- | --- | --- |
| Citrate cycle (TCA cycle) | 20 | 2 | 2.14E-02 | 1.670 | 0.100 |
| Pyruvate metabolism | 22 | 2 | 2.57E-02 | 1.591 | 0.091 |
| Glycolysis / Gluconeogenesis | 26 | 2 | 3.51E-02 | 1.455 | 0.077 |
| Alanine, aspartate and glutamate metabolism | 28 | 2 | 4.03E-02 | 1.395 | 0.071 |
| Arginine and proline metabolism | 38 | 2 | 7.00E-02 | 1.155 | 0.053 |
| Arginine biosynthesis | 14 | 1 | 1.51E-01 | 0.820 | 0.071 |
| Butanoate metabolism | 15 | 1 | 1.61E-01 | 0.792 | 0.067 |
| Purine metabolism | 65 | 2 | 1.73E-01 | 0.763 | 0.031 |
| Propanoate metabolism | 23 | 1 | 2.37E-01 | 0.625 | 0.043 |
| Galactose metabolism | 27 | 1 | 2.72E-01 | 0.565 | 0.037 |
| Glutathione metabolism | 28 | 1 | 2.81E-01 | 0.551 | 0.036 |
| Glyoxylate and dicarboxylate metabolism | 32 | 1 | 3.14E-01 | 0.502 | 0.031 |
| Glycine, serine and threonine metabolism | 33 | 1 | 3.23E-01 | 0.491 | 0.030 |
| Cysteine and methionine metabolism | 33 | 1 | 3.23E-01 | 0.491 | 0.030 |
| Amino sugar and nucleotide sugar metabolism | 37 | 1 | 3.54E-01 | 0.451 | 0.027 |
| Pyrimidine metabolism | 39 | 1 | 3.70E-01 | 0.432 | 0.026 |
| Tryptophan metabolism | 41 | 1 | 3.84E-01 | 0.415 | 0.024 |
| Tyrosine metabolism | 42 | 1 | 3.92E-01 | 0.407 | 0.024 |
| Steroid biosynthesis | 42 | 1 | 3.92E-01 | 0.407 | 0.024 |
| Primary bile acid biosynthesis | 46 | 1 | 4.20E-01 | 0.376 | 0.022 |
| Steroid hormone biosynthesis | 85 | 1 | 6.40E-01 | 0.194 | 0.012 |

^a^: Number of total metabolites in pathway; ^b^: Number of metabolites detected; ^c^: ratio of detected metabolites

in the pathway. Result from online tool (https://www.metaboanalyst.ca).

**Table S9: Pathway enriched of common differential metabolites in PDX1 and PDX2**

| **Pathway** | **Total^a^** | **Hits^b^** | **P-value** | **-log_10_P** | **Rich factor^c^** |
| --- | --- | --- | --- | --- | --- |
| Citrate cycle (TCA cycle) | 20 | 2 | 4.23E-03 | 2.374 | 0.100 |
| Alanine, aspartate and glutamate metabolism | 28 | 2 | 8.24E-03 | 2.084 | 0.071 |
| Butanoate metabolism | 15 | 1 | 7.50E-02 | 1.125 | 0.067 |
| Pyruvate metabolism | 22 | 1 | 1.08E-01 | 0.965 | 0.045 |
| Propanoate metabolism | 23 | 1 | 1.13E-01 | 0.947 | 0.043 |
| Glycolysis / Gluconeogenesis | 26 | 1 | 1.27E-01 | 0.897 | 0.038 |
| Glutathione metabolism | 28 | 1 | 1.36E-01 | 0.866 | 0.036 |
| Glyoxylate and dicarboxylate metabolism | 32 | 1 | 1.54E-01 | 0.812 | 0.031 |
| Glycine, serine and threonine metabolism | 33 | 1 | 1.58E-01 | 0.800 | 0.030 |
| Cysteine and methionine metabolism | 33 | 1 | 1.58E-01 | 0.800 | 0.030 |
| Arginine and proline metabolism | 38 | 1 | 1.80E-01 | 0.744 | 0.026 |
| Pyrimidine metabolism | 39 | 1 | 1.85E-01 | 0.733 | 0.026 |
| Tryptophan metabolism | 41 | 1 | 1.93E-01 | 0.713 | 0.024 |
| Tyrosine metabolism | 42 | 1 | 1.98E-01 | 0.704 | 0.024 |
| Purine metabolism | 65 | 1 | 2.91E-01 | 0.537 | 0.015 |

^a^: Number of total metabolites in pathway; ^b^: Number of metabolites detected; ^c^: ratio of detected metabolites

in the pathway. Result from online tool (https://www.metaboanalyst.ca).

**Table S10: Pathway enriched of differential metabolites between PDX1 and PDX2**

| **Pathway** | **Total^a^** | **Hits^b^** | **P-value** | **-log_10_P** | **Rich factor^c^** |
| --- | --- | --- | --- | --- | --- |
| Alanine, aspartate and glutamate metabolism | 28 | 2 | 2.51E-02 | 1.601 | 0.071 |
| Synthesis and degradation of ketone bodies | 5 | 1 | 4.44E-02 | 1.353 | 0.200 |
| Pyrimidine metabolism | 39 | 2 | 4.64E-02 | 1.334 | 0.051 |
| Aminoacyl-tRNA biosynthesis | 48 | 2 | 6.74E-02 | 1.171 | 0.042 |
| Arginine biosynthesis | 14 | 1 | 1.20E-01 | 0.922 | 0.071 |
| Butanoate metabolism | 15 | 1 | 1.28E-01 | 0.894 | 0.067 |
| Cysteine and methionine metabolism | 33 | 1 | 2.61E-01 | 0.583 | 0.030 |
| Glycerophospholipid metabolism | 36 | 1 | 2.81E-01 | 0.551 | 0.028 |
| Arginine and proline metabolism | 38 | 1 | 2.95E-01 | 0.531 | 0.026 |
| Purine metabolism | 65 | 1 | 4.52E-01 | 0.344 | 0.015 |

^a^: Number of total metabolites in pathway; ^b^: Number of metabolites detected; ^c^: ratio of detected metabolites

in the pathway. Result from online tool (https://www.metaboanalyst.ca).
