# Supplementary figures and images for: A novel PDX modeling strategy and its application in metabolomics study for malignant pleural mesothelioma
Source: BMC Cancer. 2021 Nov 17;21:1235. doi: 10.1186/s12885-021-08980-5 (PMC8600931; doi:10.1186/s12885-021-08980-5)

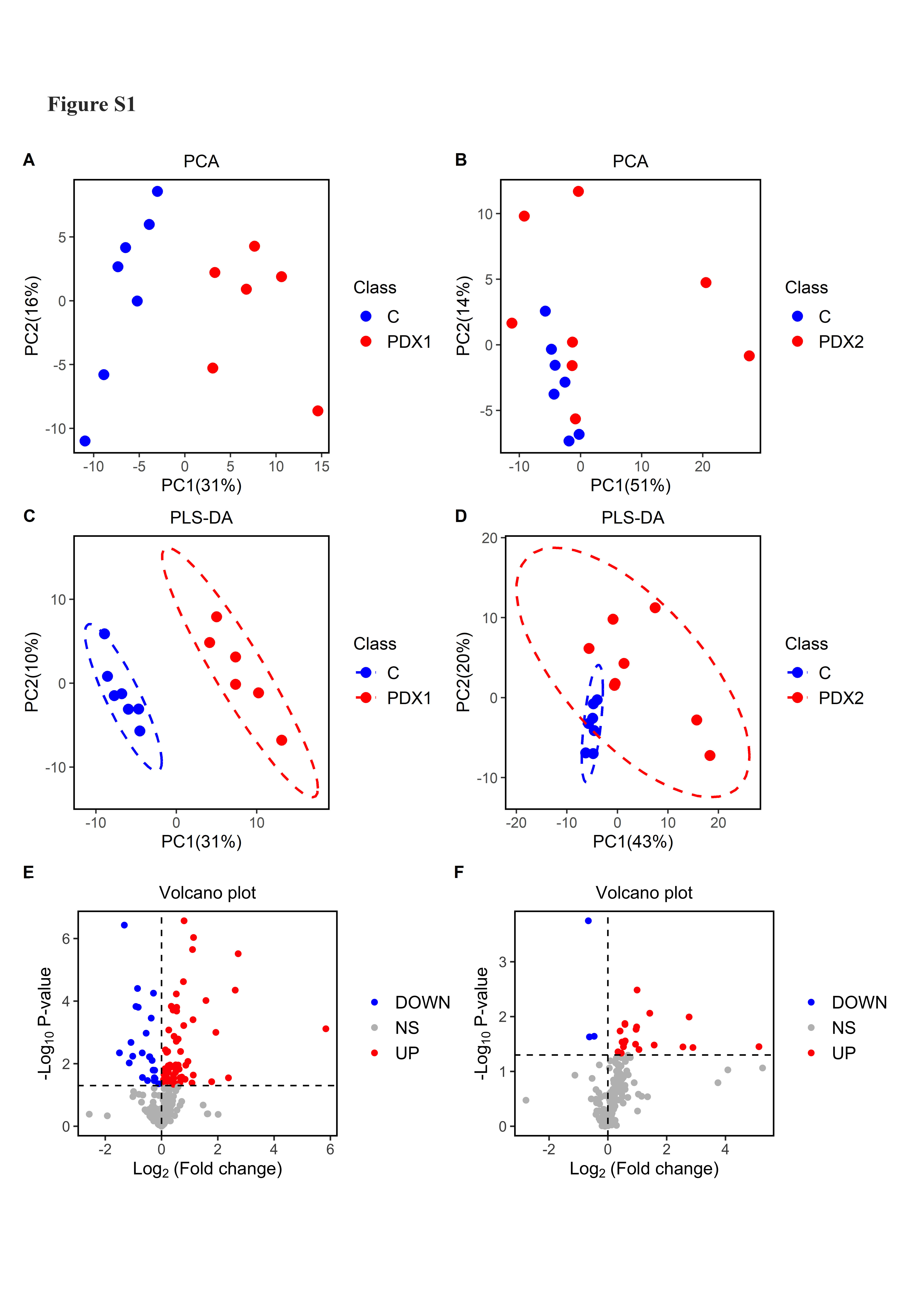

Supplement: Supplementary file 2 — Additional file 2. [file 12885_2021_8980_MOESM2_ESM.jpg]

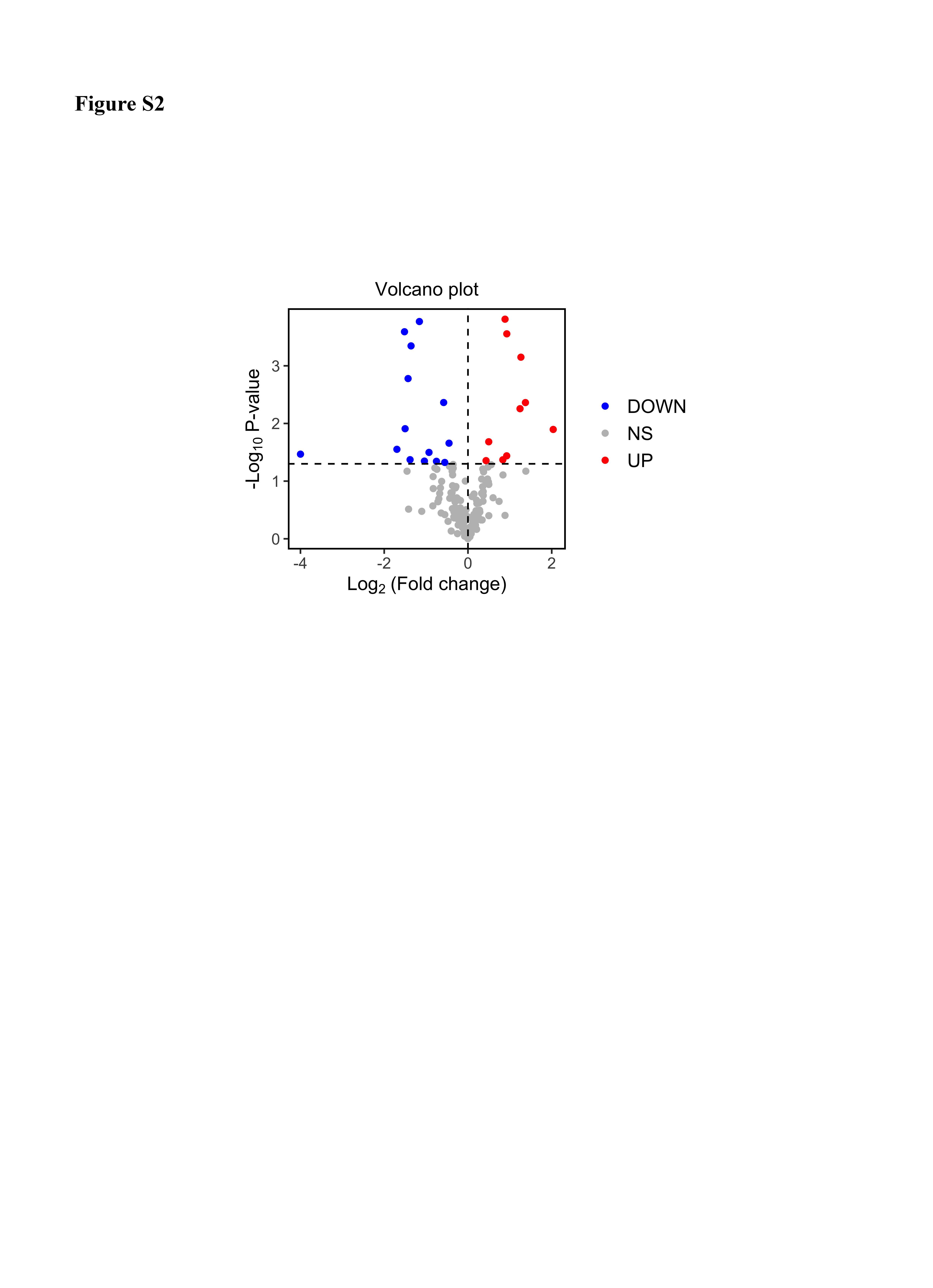

Supplement: Supplementary file 3 — Additional file 3. [file 12885_2021_8980_MOESM3_ESM.jpg]
