# Supplementary material for: A novel PDX modeling strategy and its application in metabolomics study for malignant pleural mesothelioma
Source: BMC Cancer. 2021 Nov 17;21:1235. doi: 10.1186/s12885-021-08980-5 (PMC8600931; doi:10.1186/s12885-021-08980-5)
Supplement: Supplementary file 4 — Additional file 4. [file 12885_2021_8980_MOESM4_ESM.docx]

**Supplemental information**

**Chemical regents**

Methanol (HPLC grade), water (HPLC grade), and n-hexane (HPLC grade) were purchased from ANPEL Laboratory Technologies (Shanghai) Inc. (Shanghai, China); 2-chloro-l-phenylalanine (purity>97%), pyridine (HPLC grade), methoxylamine hydrochloride (purity > 97%), Bis(trimethylsilyl)trifluoroacetamide + 1% Trimethylchlorosilane were obtained from Shanghai Aladdin Bio-Chem Technology Co.,LTD (Shanghai, China). Acetonitrile (HPLC grade) was purchased from from Tedia (Ohio, USA).

**Use of other equipment**

Ultrasonic Cleaner (SB-5200DT, Ningbo Xinzhi Biological Technology Co., Ltd.), vortex oscillator (TYXH-I, Shanghai Hannuo Instrument Co., Ltd.), Freeze-concentrated centrifugal dryer(LNG-T98, Taicang Huamei Biochemical Instrument Factory), High-speed refrigerated centrifuge (TGL-16MS, Shanghai Lu Xiangyi Instrument Co., Ltd.), Gas bath constant temperature oscillator (THZ-82A, Jiangsu Huanyu Scientific Instrument Factory), Vacuum drying oven (DZF-6021,Shanghai Huitai Co., Ltd.).

**Main antibodies used for Immunohistochemistry**

Mouse anti CK5/6 monoclonal antibody (company MXB Biotechnologies, Fuzhou, catalogue number # MAB-0744, Clone ID: D5/16B4);

mouse anti Calretinin monoclonal antibody (company Leica Biosystems, catalogue number #PA0346, Clone ID: CAL6);

Mouse anti Podoplanin (D2-40) monoclonal antibody (company Agilent DAKO, catalogue number #IR072 Clone ID: D2-40);

mouse anti WT1 monoclonal antibody (company Agilent DAKO, catalogue number #IR055); mouse anti MOC31 monoclonal antibody (company MXB Biotechnologies, catalogue number # MAB-0280, Clone ID: MOC-31);

Mouse anti paired box 8 (PAX8) monoclonal antibody (company OriGene Technologies, catalogue number #ITA327724 Clone ID: MRQ-50);

Mouse anti CAM5.2 monoclonal antibody (company MXB Biotechnologies, Fuzhou, catalogue number #MAB-0687 Clone ID: CAM5.2);

Mouse anti vimentin (VIM) monoclonal antibody (company OriGene Technologies, catalogue number #TA801250 Clone ID: OTI5D7).

**Sample preparation**

The frozen serum samples were thawed on ice, and 30 μL serum was transferred to a 1.5 mL tube. After adding 120 μL of ice-cold mixture of methanol and acetonitrile (v:v, 2:1) containing 10 μg/L  2-chloro-l-phenylalanine as internal standard, the mixture was vortexed for 1 min and then ultrasonicated for 5 min on ice. After being kept at -20 °C for 10 min, the mixture was spined at 15,000 g, 4 °C for 15 min, and 150 μL of supernatant was transferred to a glass vial for vacuum drying. Subsequently, 80 μL methoxyamine solution (methoxyamine HCl in pyridine, 15mg/mL) was added, and vortexed for 2 min and incubated at 37 °C for 90 min. Then the derivatization was initiated by adding 20 μL n-hexane and 80 μL BSTFA (containing 1% TMCS). After derivatization, the samples were kept at room temperature for 30 min before GC-MS analysis. Pooled quality control (QC) was prepared by mixing an equal amount (20 μL) from each sample, and then QCs were prepared together with the samples.

**GC-MS metabolomics analysis**

Agilent 7890B GC coupled to Agilent 5977A mass detector (Agilent Technologies Inc., CA, USA) was utilized. Separation was performed on a DB-5MS fused-silica capillary column (30 m, 0.25mm, 0.25 μm; Agilent Technologies Inc., CA, USA). Helium (purity > 99.999%) was used as carrier gas with a flow rate of 1.0 mL/min. The gradient temperatures started at 60°C, increased to 125 °C with a rate of 8 °C/min and to 210 °C with a rate of 5 °C/min, and finally to 270 °C with a rate of 10 °C/min and kept at 305 °C for 5 min.

The sample (1 μL) was loaded in a splitless injection mode with an injector temperature 260 °C. The temperatures for ion source and quadrupole were 150 °C and 230 °C, respectively. The electron energy was 70 eV. Full-scan mode (m/z 50-500) was used, and solvent delay was 5 min. QC samples were injected after every 10 samples throughout the whole analysis.
